# Vigilant: An Engineered VirD2-Cas9 Complex for Lateral Flow Assay-Based Detection of SARS-CoV2

**DOI:** 10.1021/acs.nanolett.1c00612

**Published:** 2021-04-12

**Authors:** Tin Marsic, Zahir Ali, Muhammad Tehseen, Ahmed Mahas, Samir Hamdan, Magdy Mahfouz

**Affiliations:** † Laboratory for Genome Engineering and Synthetic Biology, Division of Biological Sciences, King Abdullah University of Science and Technology (KAUST), Thuwal 23955-6900, Saudi Arabia; ‡ Laboratory of DNA Replication and Recombination, Biological and Environmental Sciences and Engineering Division, King Abdullah University of Science and Technology (KAUST), Thuwal 23955-6900, Saudi Arabia

**Keywords:** COVID-19, SARS-CoV-2, RT-RPA, lateral flow assay, CRISPR-Cas9, nucleic acid detection, VirD2, dCas9, biosensors, molecular diagnostics, relaxases

## Abstract

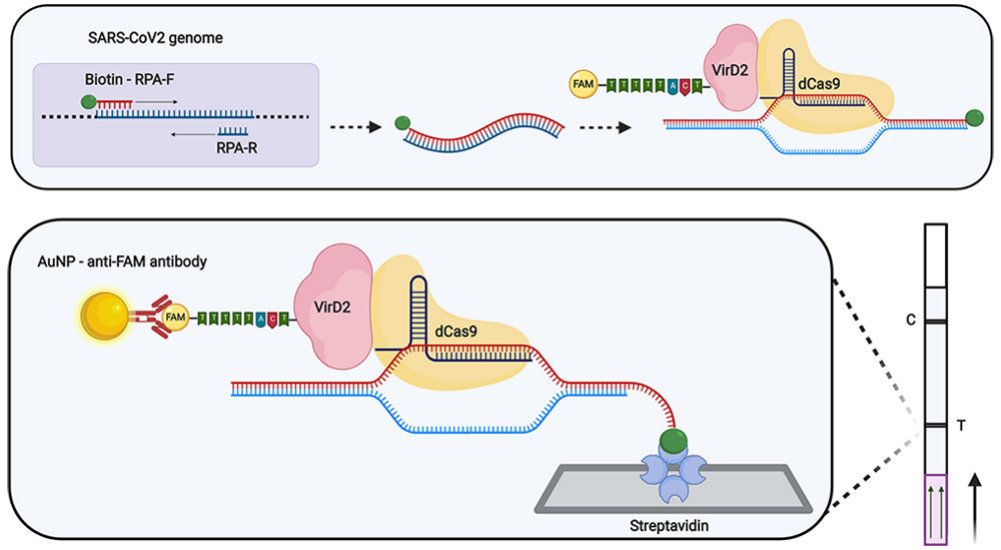

Rapid, sensitive, and specific point-of-care testing for pathogens is crucial for disease control. Lateral flow assays (LFAs) have been employed for nucleic acid detection, but they have limited sensitivity and specificity. Here, we used a fusion of catalytically inactive SpCas9 endonuclease and VirD2 relaxase for sensitive, specific nucleic acid detection by LFA. In this assay, the target nucleic acid is amplified with biotinylated oligos. VirD2-dCas9 specifically binds the target sequence via dCas9 and covalently binds to a FAM-tagged oligonucleotide via VirD2. The biotin label and FAM tag are detected by a commercially available LFA. We coupled this system, named Vigilant (VirD2-dCas9 guided and LFA-coupled nucleic acid test), to reverse transcription-recombinase polymerase amplification to detect SARS-CoV2 in clinical samples. Vigilant exhibited a limit of detection of 2.5 copies/μL, comparable to CRISPR-based systems, and showed no cross-reactivity with SARS-CoV1 or MERS. Vigilant offers an easy-to-use, rapid, cost-effective, and robust detection platform for SARS-CoV2.

## Introduction

Rapid, sensitive, and specific diagnostics can detect pathogens and disease markers in humans, animals, plants, water, and the environment,^1,2^ thus aiding treatment and mitigation measures. Although PCR-based and other sequence-based laboratory tests are capable of specific and sensitive detection of nucleic acids, they cannot meet the increasing demand for diagnostics, due to major drawbacks including their cost, turnaround time, the limited number of samples that can be processed, and the need for sophisticated equipment and skilled technical personnel.^3^ Because of the widespread applications and the promise to improve human life, there is a pressing need for the development of point-of-care (POC) or at-home testing kits capable of detecting the presence of disease or infectious markers rapidly and with the desired sensitivity but low cost.^4^ POC testing must meet the “ASSURED” criteria by being accurate, specific, sensitive, user-friendly, rapid, equipment-free, and deliverable to end-users. These criteria have been recommended by the WHO for an effective POC test to control and manage infectious diseases, especially in epidemic or pandemic situations.^5^

COVID-19 is caused by *severe acute respiratory syndrome coronavirus 2019* (SARS-CoV2), a member of the Coronaviridae family whose members pose an ongoing, major threat to public health.^6^ PCR-based testing is the gold standard for virus detection but suffers from major drawbacks that limit its use for effective, large-scale testing in pandemic situations. Therefore, there is a pressing need to develop POC testing modalities that can be deployed for testing on a massive scale.^7^ Moreover, the availability of diagnostic platforms for broad, in-field deployment is of paramount importance in preventing the further spread of COVID-19 and future pandemics.^2,4^

Clustered regularly interspaced short palindromic repeats (CRISPR) and CRISPR-associated protein (Cas) systems have been harnessed for gene editing, and the nuclease-dead mutants (dCas9) have been used for gene regulation across diverse species.^8−12^ Recently, CRISPR systems have been harnessed for diagnostics. CRISPR-Dx relies on the ability of the CRISPR system to scan the nucleic acid and find a complementary sequence to the single-guide RNA of the CRISPR complex to activate the *cis* and *trans* activities of the CRISPR enzyme. Cas13, Cas12, and Cas9 enzymes have been used to develop different CRISPR-based modalities, including SHERLOCK, DETECTR, iSCAN, SHINE, and CASLFA.^1,13−17^ These systems rely on the trans collateral activity of the CRISPR enzymes after the cis activation upon binding to the target sequence. There is a pressing need to develop CRISPR systems that complement existing detection methods, thereby expanding the power and applications of the CRISPR enzymes in diverse modalities for nucleic acids diagnostics.^18^

Relaxases are bacterial enzymes that catalyze a site- and DNA-strand-specific cleavage and help to pilot the transfer of DNA across bacterial cells or other species. Upon *Agrobacteriumtumefaciens infection*, a relaxosome complex of VirD1 and VirD2, binds to the Ti plasmid, and VirD2 cleaves the bottom strand of the Ti plasmid in the left and right borders. Interestingly, VirD2 remains covalently bound to the 5′ end of the single-stranded T-DNA through tyrosine 29.^19,20^ This property proved to be a useful tool for genome engineering.^21−24^ Here, we hypothesized that the single-stranded DNA-binding activity of VirD2 might improve the detection of nucleic acids by enabling the visualization of a Cas9–DNA complex.

Lateral flow assays (LFAs) have played critical roles in diagnostics, but the extraordinary potential of LFAs has not yet been fully exploited for widespread use in the detection of different analytes from diverse sources.^25−28^ Although LFAs have been developed to detect nucleic acids, the current technologies detect the presence or absence of the target sequences irrespective of their identity or the authenticity of the sequence. For example, recombinase polymerase amplification (RPA) coupled with LFA was used for nucleic acid detection, but the primers and primer dimers could cause false positives, compromising the specificity of the assay; moreover, the addition of the expensive specificity reagent to LFAs makes it unfeasible for massive testing.^29^ Therefore, there is a pressing need to develop a simple, inexpensive, sensitive, and specific LFA for nucleic acid detection, which can be employed for virus detection, including SARS-CoV2. Building CRISPR-based LFAs for virus detection, including SARS-CoV2, is of paramount importance to help control the pandemic.

Here, we designed, built, and tested a modality harnessing the dual functions of the CRISPR/Cas9 enzyme for DNA scanning and recognition and the VirD2 relaxase for covalent binding to a single-stranded DNA (ssDNA) probe, coupled with a LFA for virus detection. To this end, we employed a chimeric fusion between dCas9 and VirD2 coupled with a ssDNA reporter as a detection complex. Our data show that the Vigilant system provides a sensitive, specific, and low-cost modality for COVID-19 detection and nucleic acid detection in general, which can be employed as a POC test.

## Results

### Design and Construction of Vigilant for Nucleic Acid Detection

We hypothesized that a fusion of SpCas9 or SpdCas9 and VirD2 could be exploited to develop a detection platform by harnessing the unique properties and characteristics of each protein. VirD2-Cas9 fusion protein can remain bound to specifically designed ssDNA sequences; this led us to hypothesize that a short oligonucleotide with FAM at its 3′-end could remain covalently attached to the Tyr29 after VirD2-dCas9 recognition.^14^ To demonstrate that VirD2 in the fusion protein can successfully bind a 3′-end-labeled ssDNA of interest (Supporting Information Figure 1), we incubated a ssDNA consisting of a specific 25-bp VirD2 recognition sequence and a 5-T nucleotide bridge with a biotin label at the 3′ end. Western blot analysis detected the presence of the biotin-labeled moiety attached to the protein, thus confirming the activity of VirD2 protein in the fusion construct (Supporting Information Figure 2).

To confirm the ssDNA binding ability of our fusion modules, we performed gel mobility shift assays. Incubation of a 64-bp ssDNA (harboring the T-DNA right border sequence) with VirD2-Cas9 fusion proteins demonstrated a mobility shift of the VirD2-Cas9–DNA complex (Supporting Information Figure 3). The results demonstrated that the fusion modules in both orientations can bind ssDNA; however, a different shift pattern for Cas9-VirD2 was observed. We speculate that this different shift might be due to steric interferences that arise due to the subunits’ spatial orientation. Moreover, we showed that the Cas9 part of the complex retains its enzymatic activity of binding and cleaving target DNA (Supporting Information Figure 4). Therefore, each component of the chimeric fusion is fully active.

Subsequently, we hypothesized that the resulting complex could be used as a reporter to detect isothermally amplified, biotin-labeled amplicons. In the proposed module, Cas9 provides target-specific binding and VirD2 carries a 3′ FAM-labeled oligonucleotide probe for detection. To assemble the required components, we designed FAM-labeled oligonucleotides that contained a 25-nt VirD2 recognition sequence at the 5′ end and a short sequence containing five or ten thymines, followed by FAM at the 3′ end. VirD2 cleaves in the recognition sequence and remains covalently bound to the short oligonucleotide probe labeled with FAM at its 3′ end.

**Figure 1:**
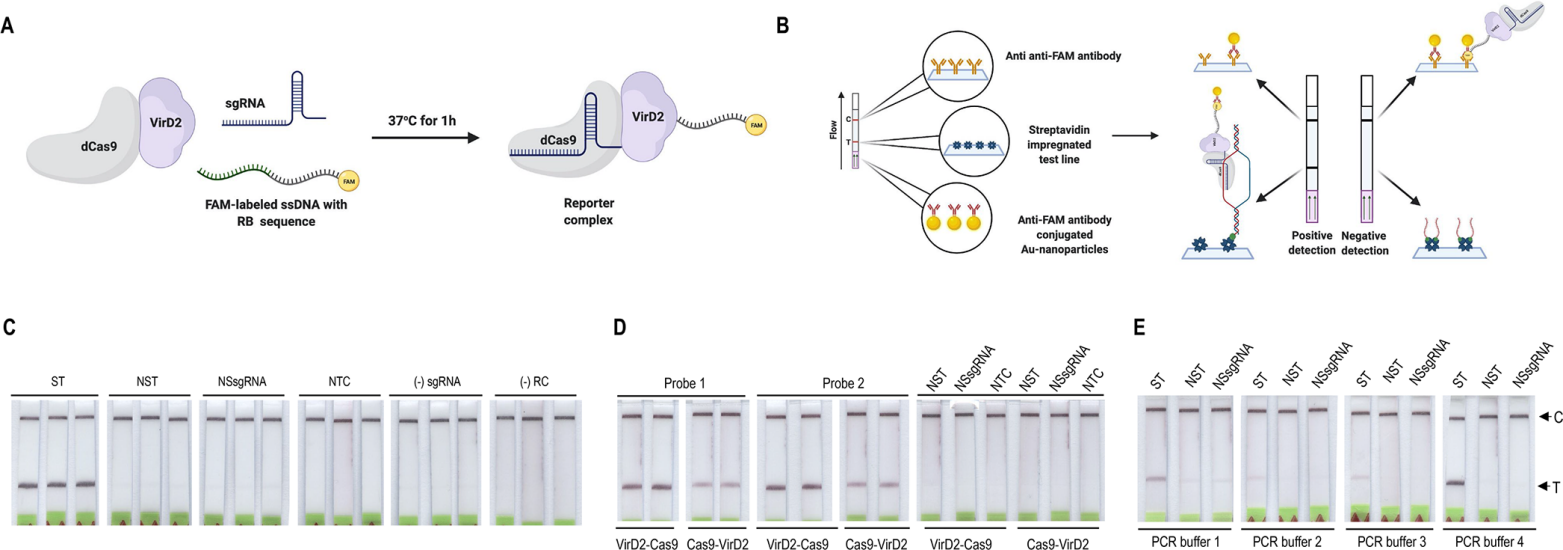
Vigilant platform for nucleic acid detection. (A) Schematic of reporter complex (FAM-probe–VirD2-dCas9–sgRNA) assembly. The ssDNA probe consists of a 25-bp T-DNA right border VirD2 recognition site at the 5′ end and a 5-bp dT stretch with FAM at its 3′ end. VirD2 recognizes the specific 25-bp motif in the ssDNA probe, cleaves it, and remains covalently bound to the 5′ end of the ssDNA probe. dCas9 uses the specific sgRNA to target the SARS-CoV-2 *N* gene. (B) Lateral flow assay. The streptavidin-coated line (T) captures biotin-labeled amplicons. In a sample containing SARS-CoV-2, the reporter complex bound to the target DNA will accumulate gold (Au) nanoparticle-labeled anti-FAM, resulting in the visual detection at the test line. The control line (C) is impregnated with anti-anti FAM antibodies that also accumulate Au nanoparticle-labeled anti-FAM and thus serve as a positive control. Detection of the positive samples is achieved by running the lateral flow after amplification and incubation with the reporter complex. The positive reaction is indicated by the presence of the lower (test) band while the upper band represents the control band. (C) Proof-of-concept of the Vigilant platform. SARS-CoV-2 *N* gene PCR amplicons were used as detection targets. PCR product (5 μL) was challenged with the preassembled reporter complex. ST (specific *N*-gene target), NST (nonspecific *N*-gene target), NSsgRNA (nonspecific sgRNA), NTC (no target control), (−) sgRNA (no sgRNA control), and (−) RC (no reporter complex). (D) Selection of the optimal fusion and probe. PCR product (5 μL) was challenged with the preassembled reporter complexes made with VirD2-Cas9 or Cas9-VirD2. Two probes, having 5 T nucleotides (probe 1) and 10 T nucleotides (probe 2) were used in the assembly of the reporter complex. Reactions with no sgRNA, no target control, or unlabeled target were used as controls. ST (specific *N*-gene target), NST (nonspecific *N*-gene target), NSsgRNA (nonspecific sgRNA), NTC (no target control). (E) Buffer composition optimization. Four buffers, having different compositions (see Materials and Methods in the Supporting Information) were used. Buffer 4 with BSA added to the running buffer was selected based on the enhanced signal detection and lower nonspecific background. ST (specific *N*-gene target), NST (nonspecific *N*-gene target), NSsgRNA (nonspecific sgRNA).

Next, we designed a sgRNA to target the SARS-CoV-2 *N* gene. sgRNA complementarity will bring the VirD2-dCas9-ssDNA-FAM to the *N* gene sequence. As the final part of the complex, we designed biotin-labeled primers to amplify the target DNA, which was reverse-transcribed and amplified from the SARS-CoV-2 *N* gene via polymerase enzyme. When these components are assembled, the resulting structure (Biotin-DNA + VirD2-Cas9-sgRNA-ssDNA-FAM) is both biotin and FAM-labeled and can therefore be detected using commercially available lateral flow strips (Figure 1A and B).

### Vigilant Specifically Detects Nucleic Acids

Next, we performed a proof-of-principle detection assay with amplicon targets generated by PCR, targeting the *N* gene of the SARS-CoV-2 genome. The reporter complex consisting of the VirD2-Cas9 fusion protein, sgRNA, and FAM-labeled ssDNA oligonucleotide was preassembled at 37 °C for 1 h. Following amplification of the *N* gene, 5 μL of the unpurified, biotin-labeled PCR product was transferred to the reaction containing preassembled reporter complex, and the mixture was incubated at 37 °C for 10 min to assemble the sgRNA-VirD2-Cas9-ssDNA-FAM complex and at 60 °C for 1 min to release any nonspecifically bound DNA.

Following the addition of the running buffer, the reaction was applied to the lateral flow strip and the band at the test line appeared within 3 min specifically in the samples containing the correct amplicons (Figure 1C). To test the parameters for our reporter system, we assembled the complex using VirD2 and Cas9 in both orientations (VirD2-Cas9 or Cas9-VirD2) and using probes containing short (five-T) and long (ten-T) spacers. The experimental results indicate that VirD2-Cas9 with both short and long spacer probes displays superior performance compared to Cas9-VirD2 (Figure 1D).

To further optimize the system for maximum performance with PCR products, we tested various buffer compositions. PCR buffer 4 showed better results than the other buffers used (Figure 1E). This demonstrates that the buffer composition plays a major role in the performance of the assay. We optimized the reaction conditions for VirD2-Cas9 for specific and sensitive detection of nucleic acids.

### Vigilant Demonstrates Robust Detection of RT-RPA Products

**Figure 2:**
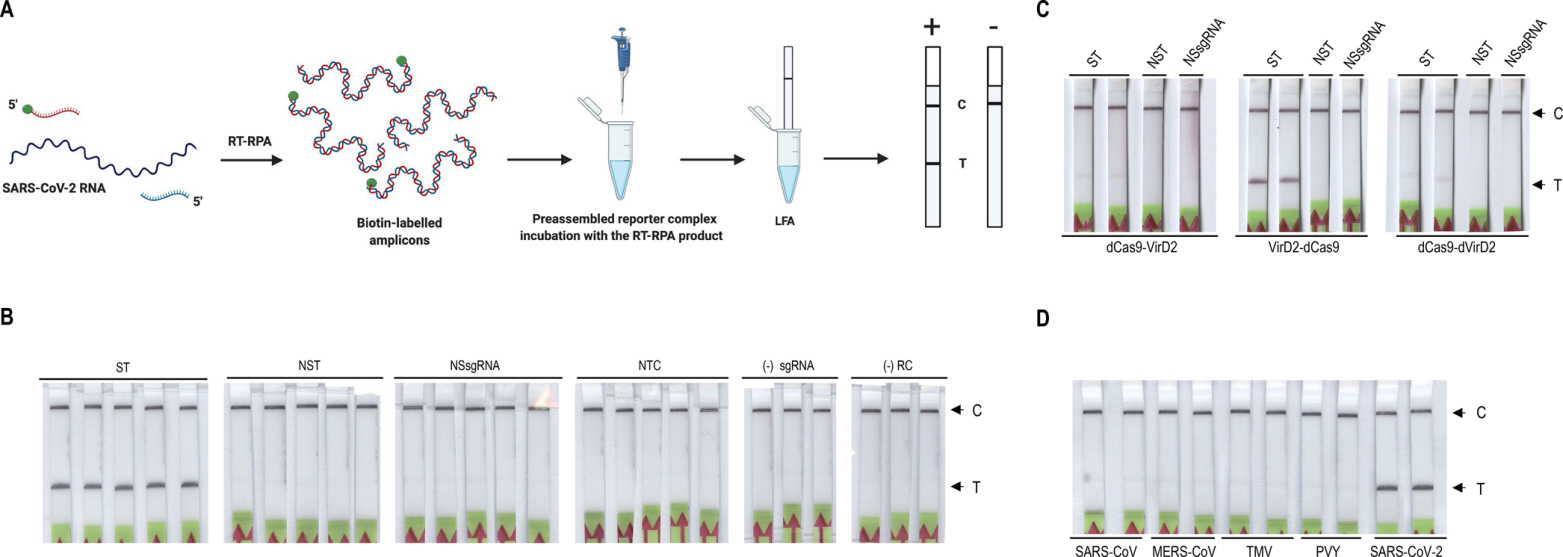
RT-RPA coupled with Vigilant for SARS-Cov2 detection. (A) SARS-CoV-2 RNA is reverse transcribed and amplified by RT-RPA using biotin-labeled primers. The biotin-labeled amplicon is then mixed with the preassembled reporter complex. Upon target recognition via the sgRNA, Cas9 remains stably bound to the DNA, yielding a complex that is labeled with both FAM (probe) and biotin (target). (B) RT-RPA product detection with the Vigilant platform. SARS-CoV-2 (synthetic) *N*-gene RT-RPA amplified product used as detection target. RT-RPA product (5 μL) was challenged with the preassembled reporter complex. Nonspecific *N*-gene target and nonspecific sgRNA, no target control, no sgRNA, and no reporter complex were used as controls. ST (specific *N*-gene target), NST (nonspecific *N*-gene target), NSsgRNA (nonspecific sgRNA), NTC (no target control), (−) sgRNA (no sgRNA control), and (−) RC (no reporter complex). (C) The Vigilant platform is compatible with VirD2-dCas9. Fusion proteins, VirD2-dCas9, dCas9-VirD2, and dCas9-dVirD2 were purified and evaluated for the Vigilant platform. RT-RPA product (5 μL) was challenged with the preassembled reporter complexes made with VirD2-dCas9 or dCas9-VirD2 or dCas9-dVirD2. VirD2-dCas9 demonstrated superior performance compared to the other two fusion proteins. ST (specific *N*-gene target), NST (nonspecific *N*-gene target), NSsgRNA (nonspecific sgRNA). (D) The Vigilant platform specifically detected SARS-CoV-2. RT-RPA was performed using SARS-CoV, SARS-CoV-2, MERS-CoV, TMV, and PVY as templates. RT-RPA product (5 μL) was challenged with the preassembled reporter complex. The Vigilant platform specifically detected only SARS-CoV-2.

Due to its simplicity and rapidity, RPA has been widely used for the detection of SARS-CoV-2 coupled to LFAs via Cas12 and Cas13 CRISPR-based detection methods. We therefore tested whether the Vigilant system is capable of detecting RT-RPA products (Figure 2A). To this end, we designed four sets of RPA primers each targeting two different regions within the SARS-CoV-2 nucleocapsid (*N*) gene and screened them for optimal amplification performance (Supporting Information Figure 5). After selecting the best-performing set, 5 μL of the RPA amplification mixture was exposed to the preassembled reporter complex. The experimental results yielded a positive result at the test line on the lateral flow strips in the reactions containing only the correct amplicons while showing no bands at the test line in control reactions, including the nonspecific amplicon of SARS-CoV-2 *N*-gene (Figure 2B). RT-RPA reactions were further optimized by screening for the most effective reaction buffer, reporter complex concentration, RT-RPA input volume, and assay time (Supporting Information Figure 6). To confirm that Vigilant is compatible with the temperature required for most of the reverse transcriptases, we performed RT-RPA at 42 °C (Supporting Information Figure 7).

Inactivation of the Cas9 nuclease catalytic activity does not impair its DNA binding activity, as binding to the target depends on sgRNA complementarity rather than cleavage.^30^ We made fusions of dCas9 with VirD2 and deactivated VirD2 (dVirD2) and compared their activity with VirD2-Cas9 for target detection (Supporting Information Figure 8). Subsequently, we tested the VirD2-dCas9 module in the RT-RPA assays. The VirD2-dCas9 module demonstrated a robust detection of the SARS-CoV2 *N* gene RT-RPA product compared to the other two modules (Figure 2C). Our data show that VirD2-dCas9 is capable of specific nucleic acid detection.

Next, in order to demonstrate the specificity of Vigilant, MERS-CoV and SARS-CoV2 *N* gene template DNAs were used as controls. We also used a plant RNA virus as control. The Vigilant assay showed no cross-reactivity with the nonspecific targets and specifically detected only SARS-CoV-2 RNA (Figure 2D).

### The Vigilant Module Possesses High Sensitivity and Stability

**Figure 3:**
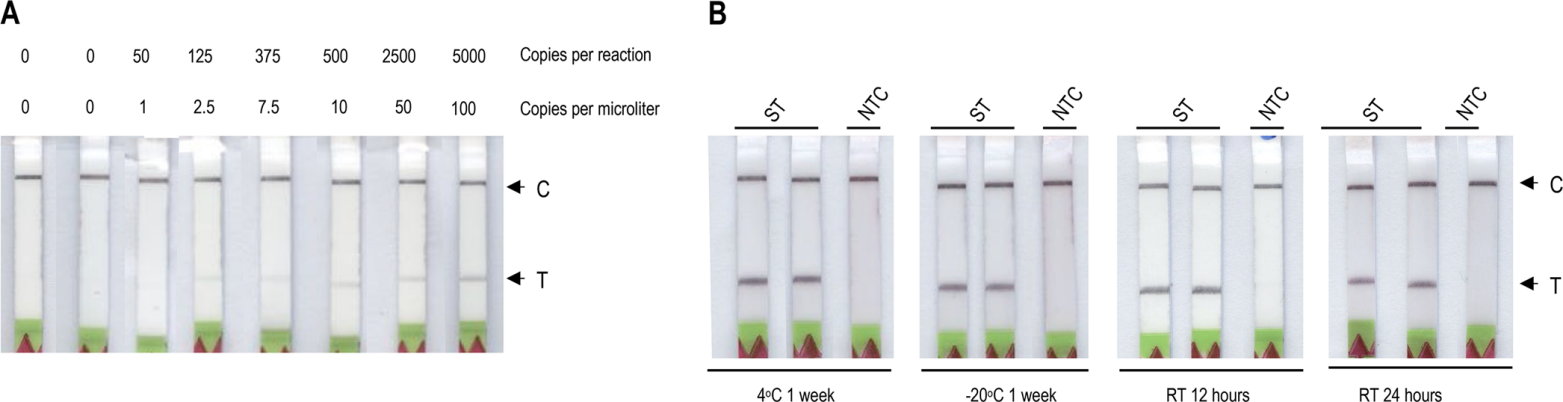
Sensitivity and stability of the Vigilant platform for nucleic acid detection. (A) LoD determination with synthetic SARS-CoV2 RNA. A serial dilution (0, 1, 2.5, 7.5, 10, 50, and 100 copies per microliter) of SARS-CoV-2 synthetic RNA was prepared and amplified by RT-RPA using biotin-labeled primers. The biotin-labeled amplicon is then challenged with the preassembled reporter complex and visualized on LFA strips. (B) Stability of the preassembled Vigilant reporter complex. The reporter complex was stored at 4 °C, −20 °C, and room temperature for different time durations. SARS-CoV-2 (synthetic) *N*-gene RT-RPA amplified product (5 μL) was then used for detection. ST (specific *N*-gene target), NTC (no target control).

Assay sensitivity is an essential characteristic for any detection platform. To determine the limit of detection (LoD) of the Vigilant system for nucleic acid detection, we tested dilutions of 0, 1, 2.5, 7.5, 10, 50, and 100 copies/μL of synthetic SARS-CoV-2 genomic RNA in the RT-RPA reaction. Mixing of the RT-RPA amplified product of each dilution with the reporter complex determined the LoD as 2.5 copies/μL (Figure 3A and Supporting Information Figure 9). For comparison, we measured the Ct values of the serial dilutions of the synthetic RNA to determine the clinical relevance of the LoD for the detection of clinical samples by RT-qPCR (Supporting Information Figure 10). Our assays showed that Vigilant could detect as little as 2.5 copies/μL of the synthetic RNA, which corresponds to 40–60 copies/reaction of virus, a concentration that is clinically relevant.^31^

Next, we evaluated the stability of the reporter complex to evaluate shelf life and storage requirements. We preassembled the reporter complex and stored it directly in the reaction buffer at room temperature for 6, 12, and 24 h. Additionally, we stored the preassembled reporter complex at 4 °C and −20 °C for 24 h, 48 h, and 1 week. After performing the detection reactions with stored reagents, we observed no decrease in performance, indicating that the reagents can be stored at room temperature in an aqueous solution at 4 °C or −20 °C for at least 1 week (Figure 3B and Supporting Information Figure 11).

### Vigilant Validation in COVID-19 Clinical Samples

**Figure 4:**
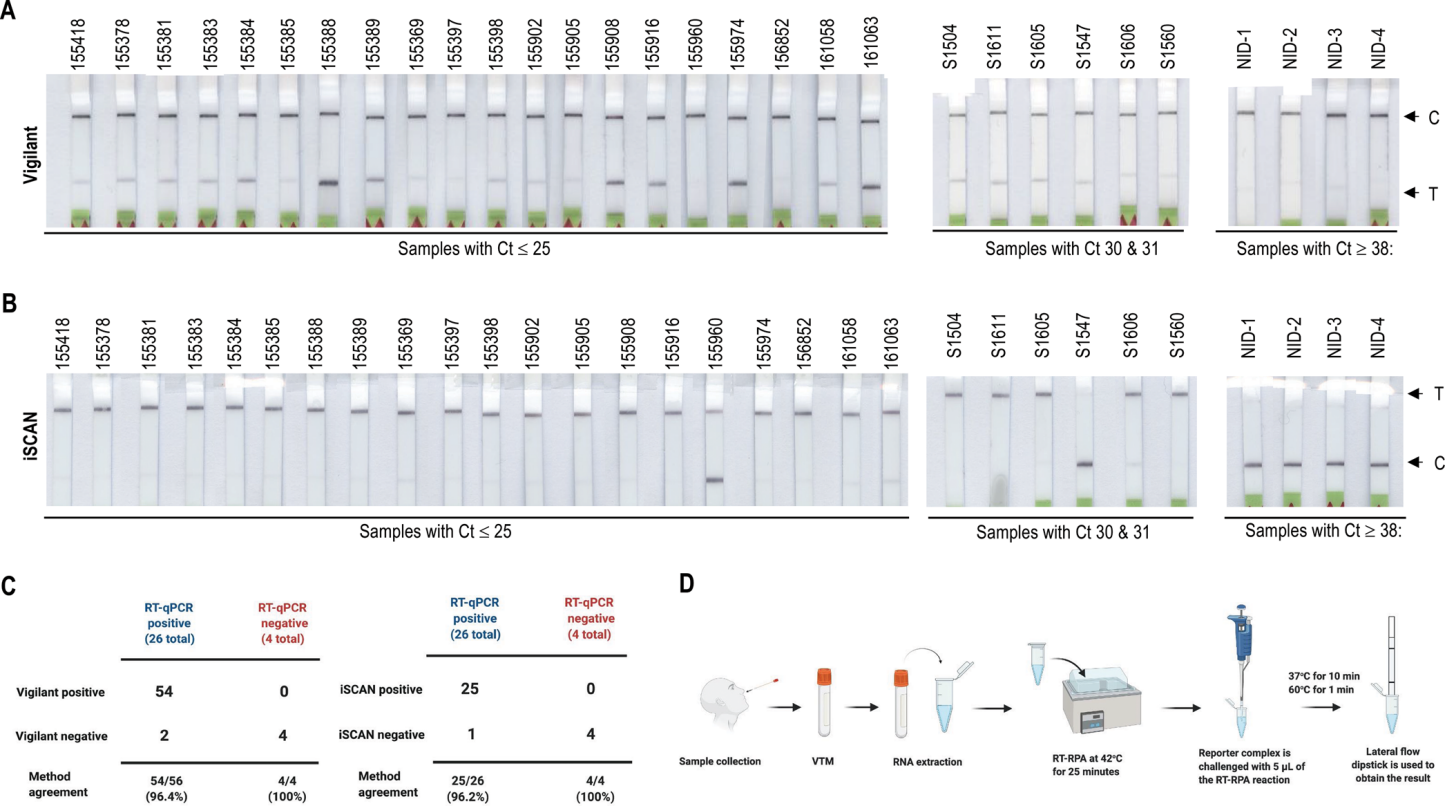
Validation of the Vigilant platform for SARS-CoV2 clinical samples. (A) Detection of SARS-CoV-2 in clinical samples. RT-RPA was performed for detection of SARS-CoV-2. SARS-CoV-2 RNA was isolated with the Trizol method. Samples with viral load (Ct value, 16–38) were detected with the Vigilant platform. The *N*-gene RT-RPA amplified product (5 μL) was subjected to the preassembled reporter complex. Samples with Ct value >38 were considered as negative. (B) Table representing experimental comparison of Vigilant, iSCAN, and RT-qPCR. (C) iSCAN method for the detection of SARS-CoV2 in clinical samples. An RT-LAMP-based iSCAN was performed for comparison with the Vigilant platform. (D) Schematic of POC utility of the Vigilant platform. After sample is collected from saliva or nasopharyngeal swab and transported in VTM, RNA is extracted and used as input for detection using Vigilant. In the first step RT-RPA with biotin-labeled primer is performed to amplify the viral genome. The resulting amplicons are then detected using the preassembled reported complex and visualized using LFA strips.

Next, we validated Vigilant for the detection of SARS-CoV-2 in clinical samples. We optimized the detection of the signal in clinical samples by optimizing the input RNA concentration and the amount of the RT-RPA added to the Vigilant detection complex. To exclude any sample bias, we used SARS-CoV2 clinical samples with a wide range of Ct values. To avoid experimental bias, we randomized the positive and negative samples, recorded the Vigilant results, and compared these to the RT-qPCR data. We conducted the validation using 26 positive samples and 4 negative samples based on RT-qPCR (Supporting Information Table 1). Our data show that Vigilant exhibits high sensitivity, detecting 54 out of 56 positive samples. Moreover, 4 out of 4 negative samples were also negative by Vigilant. This indicates a 96.4% sensitivity and 100% specificity in agreement with RT-qPCR positive and negative samples, respectively (Figure 4A and B and Supporting Information Figure 12).

Previous reports have indicated that the use of LFA for samples with Ct values > 32 was not reproducible compared with fluorescent detection, where samples with Ct values up to 35/36 could be detected. We compared our Vigilant system with the CRISPR-Cas12-based iSCAN system in SARS-CoV-2 clinical samples. Our data show that iSCAN (lateral flow readout) detected 25/26 positive samples and all 4/4 negatives were in agreement with RT-qPCR, and Vigilant detected 54/56 positives and 4/4 negatives in agreement with qPCR (Figure 4B and C). Therefore, the Vigilant LFA system exhibits good concordance, including sensitivity and specificity, with RT-qPCR and CRISPR-Cas12-based detection systems and offers key features essential for effective POC testing (Figure 4D).

## Discussion

In this work, we developed Vigilant, an LFA that uses a fusion of VirD2 and dCas9 for nucleic acid detection. Vigilant can be used as a simple, affordable, and robust virus detection platform by coupling the system with RT-RPA reactions. In this work, we employed Vigilant for SARS-CoV2 detection in clinical samples, but it can be reprogrammed to detect any user-defined nucleic acid sequence.

Current methods involving direct coupling of the LFA to amplified nucleic acid exhibited high rates of false positives due to the formation of primer dimers and nonspecific binding of the probe reporter;^16,29^ frequent false positives also can arise from cross-contamination and nonspecific amplification. Recent efforts have attempted to address these issues by introducing an additional level of specificity, by exploiting the ability of Cas9 to remain tightly bound to its target for hours after cleavage^16,32^ . This insight inspired the development of LFA-based detection platforms called CASLFA and FELUDA, which combine isothermal amplification of the target nucleic acid with the DNA recognition and unwinding activity of Cas9.^30,33,34^ The resulting product can be detected on a specially designed lateral flow strip by using specific hybridization probes immobilized on gold nanoparticles.^16,32^ However, these platforms require complicated reagents that are uncommon in most laboratories, difficult, laborious, and expensive to prepare, as well as custom-made reporters or lateral flow strips.

To overcome these drawbacks, we designed, built, and tested the Vigilant detection system, which couples the functions of the CRISPR-Cas9 and DNA relaxases for a robust LFA for virus detection that can be field deployed for POC applications. Cas9 helicase activity eliminates the need for the DNA denaturation step, which is required in conventional hybridization-based LFAs. Vigilant provides critical features including short running time, compatibility with quick extraction protocols, and isothermal amplification, which make it a practical method to detect viruses and pathogens. The low cost, estimated at $10/reaction, makes it affordable and deployable in low-resource settings for large-scale screening of COVID-19 cases.

Clinical studies suggest that the risk of SARS-CoV-2 transmission decreases dramatically when the number of viruses drops below 1000 particles /μL.^35−37^ A recently developed model of mass pandemic surveillance suggests that assays with fast turnaround time able to detect 100 copies/μL would be adequate for efficient high-throughput screening.^38^ The low LoD offered by Vigilant surpasses this limit and is comparable to conventional PCR-based methods and newer CRISPR-based approaches. Vigilant reaction takes around 35 min with a preassembled reporter complex, which makes it ideal for both in-field and POC applications.

Vigilant introduces a new class of CRISPR-based detection that provides critical features for powerful POC detection systems that do not rely on the *trans*, collateral, or *cis* activities of CRISPR enzymes.^3,39,40^ The Vigilant principle could potentially be applied not only to Cas9 variants but also to Cas12 and Cas14 variants, where chimeric fusions between a CRISPR enzyme and a relaxase can provide the dual functions of specific binding to the target sequence and binding to a ssDNA probe resulting in a molecular complex for LFA detection. Moreover, we envision the use of the PAM-independent Cas9 variants capable of the recognition of any DNA sequence in a PAM-independent manner. This will bypass the need to find a PAM sequence for the binding site and expand the utility of this platform to any nucleic acid sequence. Similarly, other relaxases with different recognition sequences can be employed for binding to a ssDNA probe. We envision the generation of LFA based on the Vigilant principle employing different CRISPRs and relaxases. Despite significant advantages, several improvements can be made in the future to enhance the performance of the Vigilant platform. Coupling of Vigilant to a quick extraction protocol can further reduce the assay duration time since RNA extraction remains the most time-consuming and cumbersome step.^1,41^ At present, the Vigilant detection assay relies on the separation of preamplification and detection steps to avoid cross-contamination. Good practices and caution are necessary to avoid false-positive results. Recently, a CRISPR/Cas VI (AapCas12b)-based detection system (STOPCovid.v2) was applied to detect SARS-CoV2 in a one-pot assay.^41^ Such modules demonstrate high sensitivity, specificity, and ease of use that enable POC utilization of CRISPR/Cas-based diagnostics. In this work, we demonstrate that Vigilant can complement the existing CRISPR-based methods and provide comparable levels of sensitivity and specificity.

In conclusion, Vigilant and its detection principle are suitable for in-field large-scale screening and promise to advance POC nucleic acid diagnostics at a massive scale in low resource settings and to serve in controlling, managing, and mitigating the effects of COVID-19 or future pandemics.

## Supporting Information Available

Materials and methods, information on the probe sequence, gel binding assays, chimeric protein fusion sequences, and additional lateral flow assays (PDF)
