# Supplementary material for: Vigilant: An Engineered VirD2-Cas9 Complex for Lateral Flow Assay-Based Detection of SARS-CoV2
Source: Nano Lett. 2021 Apr 12;21(8):3596–603. doi: 10.1021/acs.nanolett.1c00612 (PMC8056947; doi:10.1021/acs.nanolett.1c00612)
Supplement: Supplementary file 1 — nl1c00612_si_001.pdf [file nl1c00612_si_001.pdf]

Vigilant: An engineered VirD2-Cas9 complex for lateral flow assay-based detection of SARS-CoV2

Tin Marsic[1], Zahir Ali[1], Muhammad Tehseen[2], Ahmed Mahas[1], Samir Hamdan[2], and Magdy Mahfouz[1, *]

[1] *Laboratory for Genome Engineering and Synthetic Biology, Division of Biological Sciences, 4700 King Abdullah University of Science and Technology, Thuwal 23955-6900, Saudi Arabia.*

[2] *Laboratory of DNA Replication and Recombination, Biological and Environmental Sciences and Engineering Division, King Abdullah University of Science and Technology (KAUST), Thuwal 23955-6900, Saudi Arabia.*

***Correspondence**: Magdy M. Mahfouz (magdy.mahfouz@kaust.edu.sa)

Key words: COVID-19, SARS-CoV-2, RT-RPA, lateral flow assay, CRISPR-Cas9, nucleic acid detection, VirD2, dCas9, biosensors, molecular diagnostics, relaxases

MATERIALS AND METHODS

Nucleic acid preparation

a) Plasmids, ssDNA probe, and oligos

VirD2-SpCas9 and SpCas9-VirD2 clones were used to purify the fusion proteins. Mutant VirD2-SpdCas9 (dead SpCas9-VirD2), SpdCas9-VirD2 (VirD2-dead SpCas9), and SpdCas9-dVirD2 (dead SpCas9-dead VirD2) clones for expression of the respective fusion proteins were custom synthesized by GenScript. Guide RNAs for SpCas9 experiments were designed using SnapGene and ordered as gBlocks from Integrated DNA Technologies under the T7 promoter for *in vitro* transcription. The FAM-labeled ssDNA probe and biotin-labeled oligos were ordered from Integrated DNA Technologies. Sequences of the plasmids and oligos are listed in the supplementary file.

b) *In vitro* transcription of sgRNA

In vitro transcription was performed using TranscriptAid T7 High Yield Transcription Kit (Thermo Scientific) following the manufacturer's instructions. Briefly, 10 µL of 5X TranscriptAid Reaction Buffer, 20 µL NTP mix, 10 µL of the DNA template (annealed sgRNA gBlock and T7 promoter oligo), 0.5 µL of RNase Out, 5 µL of TranscriptAid Enzyme Mix, and 4.5 µL of DEPC-treated water were incubated at 37°C for 8 hours. *In vitro* transcribed RNA was purified using Direct-zol RNA MiniPrep Kit (Zymo Research). Production of the proper size sgRNA fragments was confirmed on a 2% agarose gel run in Tris-Borate-EDTA buffer.

Protein purification

Protein purification was performed as previously described [13]. Briefly, a single colony of BL21(DE3) was grown in 2X-YT media and induced at 0.6 OD_{600} with 0.3 mM IPTG and incubated at 18°C for 15 h at 180 rpm. Proteins were isolated using affinity purification column and further purified by size fractionation using the ÄKTA pure system (Cytiva).

Functional characterization of in-house produced enzymes

a) SpCas9 nuclease activity assay of the fusion proteins

Target fragment (SARS-CoV-2, *N*-gene fragment) was amplified by PCR for SpCas9-based cleavage assays. Ribo-nucleoprotein particles of VirD2-SpCas9, SpCas9-VirD2, and catalytically dead mutants VirD2-SpdCas9, SpdCas9-VirD2 and SpdCas9-dVirD2 were assembled at 37°C for 10 minutes in 16.3 µL reaction consisting of 250 nM of the respective protein, 250 nM sgRNA in the cleavage buffer (10 mM Tris-HCl pH 8.0, 50 mM NaCl, 10 mM $MgCl_2$). Following the incubation, 3.7 µL containing 150 ng of the target was added into the tube and the reaction was incubated for 1 hour at 37°C. The protein was denatured at 95°C for 5 minutes, the reaction was cooled on ice for 3 minutes, and the DNA products were separated on a 2% agarose gel.

b) VirD2 and probe covalent binding assay of the fusion proteins

Biotin-labeled probe (ssDNA harboring the T-DNA right border sequence) was mixed with the fusion proteins (VirD2-SpCas9, SpCas9-VirD2, and catalytically dead mutants VirD2-SpdCas9, SpdCas9-VirD2 and SpdCas9-dVirD2) at the ratio 1 : 1 (250 nM each) V2 buffer (10 mM Tris-HCl pH 8.0, 50 mM NaCl, 10 mM $MgCl_2$). The reaction mix was incubated at 37°C for 60 minutes. Protein loading dye was added to the reaction and heated for 95°C for 3 minutes. The complex was resolved on 10% NuPAGE (Invitrogen) for 3 h at 4°C and transferred to nitrocellulose membrane for 3 h at 4°C. The membrane was immuno-blotted with anti-biotin mouse primary antibody 1:1000 (Cat. No. SC-53179, Santa Cruz) and anti-mouse secondary antibody 1:2000 (Cat. No. A3688, Sigma) and detected with chemiluminescent ECL solution (BioRad). Alternatively, for direct gel mobility shift, probe (64 bp ssDNA harboring the T-DNA right border sequence) was mixed with the fusion proteins (VirD2-SpCas9, SpCas9-VirD2, and catalytically dead mutants VirD2-SpdCas9, SpdCas9-VirD2 and SpdCas9-dVirD2) at the ratio 1 : 1 (250 nM each) V2 buffer (10 mM Tris-HCl pH=8.0, 50 mM NaCl, 10 mM $MgCl_2$). The reaction mix was incubated at 37°C for 60 minutes. Protein loading dye was added to the reaction and heated for 95°C for 3 minutes. The complex was resolved on 10% NuPAGE (Invitrogen) for 3 h at 4°C. Gel was stained with Coomassie brilliant blue stain (MBP). Gel photos were taken using Gel Doc XR (BioRad).

RT-RPA reactions with synthetic targets

RT-RPA was performed using the TwistAmp Basic kit following the manufacturer's instructions. Briefly, a well-mixed 47.5-µL sample (1 µl RNA template, 2.4 µL of 10 µM biotin-labeled forward and 2.4 µL of unlabeled reverse primers, 29.5 µL of Rehydration buffer, 0.5 µL of SuperScript IV reverse transcriptase, 1 µL of RNase H, 0.5 µL of RNase Out, 10.2 µL H_20) was added to the lyophilized RPA reaction components (TwistAmp Basic) and homogenized by pipetting. Magnesium acetate (2.5 µL of 280 mM) was added to each tube and mixed. The isothermal amplification was performed at 42°C for 25 minutes. To confirm the DNA isothermal amplification, 10 µL of the reactions were purified using QIAquick PCR Purification Kit and separated on a 1.5 % agarose gel.

VirD2-SpdCas9 reporter complex assembly

The reporter complex (final concentration of 250 nM each VirD2-SpdCas9, sgRNA and FAM-labeled reporter oligo) was prepared by combining 1.25 µL of 5 µM VirD2-SpCas9 secondary stock, 1.25 µL of 5 µM sgRNA, 1.25 µL of 5 µM FAM-labeled reporter oligo, 5 µL of 5X RPA reaction buffer (50 mM Tris-HCl pH 8.0, 500 mM KCl, 250 mM NaCl, 5 mM DTT, 50 mM $MgCl_2$, 250 mM L-arginine, and 250 mM L-glutamic acid) or 5 µL 5X PCR reaction buffer (100 mM HEPES pH 8.0, 500 mM KCl, 250 mM NaCl, 5 mM DTT, 25 mM $MgCl_2$), 0.5 µL of RNase Out and ultra-pure water to 20 µL. The reaction was incubated at 37°C for 60 minutes. Two reactions, 20 µL each, were combined in a single tube to make 40 µL preassembled reporter reaction.

VirD2-SpdCas9 detection assay and lateral flow assay

RT-RPA product (5 µL) was mixed with 40 µL of preassembled reporter. The 45-µL reaction mix was incubated at 37°C for 10 minutes followed by 1 minute at 60°C. Following the incubation, 55 µL of the running buffer (44.5 µL of the HybriDetect Assay Buffer with 10.5 µL of 10% BSA) was added directly into the reaction and mixed. Room temperature adjusted HybriDetect Dipsticks were placed into the tube containing the reaction mixture. Lateral flow strips were removed from the tube as soon as the control band appeared and the result was called

within 10 minutes. Images of the strips were taken within 25 minutes after the beginning of the LFA.

Limit of detection assay

SARS-CoV-2 RNA (synthetic RNA from IDT) was diluted to final concentrations corresponding to 1, 2.5, 7.5, 10, 50, and 100 copies/µL or 50, 125, 375, 500, 2500, and 5000 copies/reaction. The respective volume of RNA sample was added to the RT-RPA reactions. Nuclease-free water was used as the negative control. Following the RT-RPA reaction, 5 µL of the product was transferred into the reaction containing the preassembled reporter complex and the samples were detected as previously described. The detection limit was considered as the concentration that could be successfully detected within 10 minutes of the LFA assay in all three replicates.

Validation of the developed protocol with SARS-CoV-2 clinical sample

RNA samples from SARS-CoV-2 RT-PCR positive (26 clinical samples) and negative (4 clinical samples) were used for evaluation of our Vigilant protocol. RNA (4 µL) was added to the RT-RPA reaction and 5 µL of the amplified product was used for detection in the next step.

List of Supplementary Figures

Supplementary Figure 1. VirD2 alone is capable of cleaving ssDNA containing the specific RB recognition sequence.

Supplementary Figure 2. Covalent binding of VirD2 and ssDNA probe.

Supplementary Figure 3. Covalent binding of VirD2 and ssDNA.

Supplementary Figure 4. Confirmation of VirD2-SpCas9 and SpCas9-VirD2 nuclease activity.

Supplementary Figure 5. Selection of the optimal RT-RPA primer set.

Supplementary Figure 6. Selection of the optimal reaction buffer.

Supplementary Figure 7. Evaluation of the Vigilante at 42 ^{0}C.

Supplementary Figure 8. Comparison of VirD2-SpCas9 and VirD2-SpdCas9.

Supplementary Figure 9. Repeats 2 and 3 of LOD determination.

Supplementary Figure 10. Copy number determination by RT-qPCR for Ct value relevance in clinical samples.

Supplementary Figure 11. Reporter complex stability.

Supplementary Figure 12. Additional evaluated clinical samples and their original scan.

Supplementary table 1. Designation and the Ct value of the clinical samples used in this study. RT-qPCR was performed on Trizol based isolated RNA.

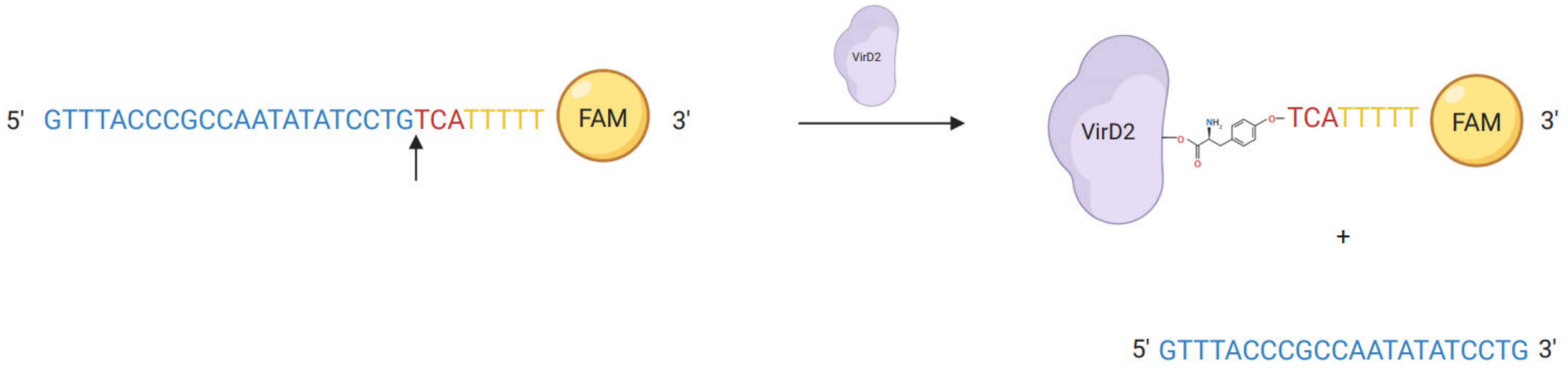

Supplementary Figure 1. VirD2 alone is capable of cleaving ssDNA containing the specific RB recognition sequence. Following the cleavage of the oligonucleotide, VirD2 remains covalently bound to Tyr29 moiety, leaving three nucleotides of the original sequence. This property can be exploited to attach labels at the 3' end of oligonucleotide sequence bound to VirD2.

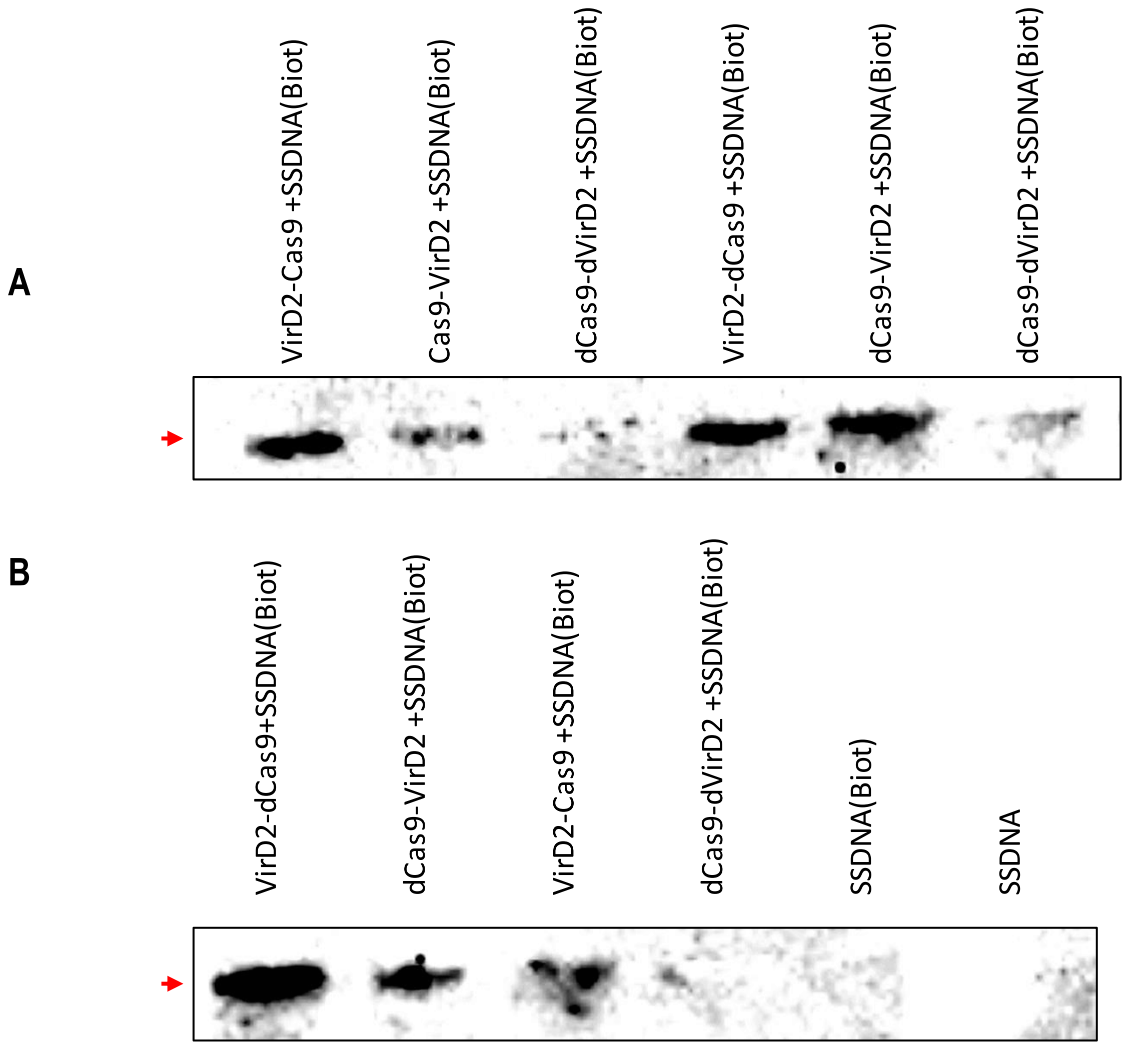

Supplementary Figure 2. A, B), Covalent binding of VirD2 and ssDNA probe. Biotin labeled probe harboring the T-DNA right border sequence was incubated with fusion proteins in the presence of Mg^{2+}. VirD2-Cas9, Cas9-VirD2, VirD2-dCas9, dCas9-VirD2 bound to biotin labeled probe were detected by western blot. Biotin labeled probe, unlabeled probe, and dCas9-dVirD2 (no binding to RB sequence containing probe) were used as experimental control. Red arrow head indicated the immuno-detection of the biotin labeled probe bounded to fusion proteins.

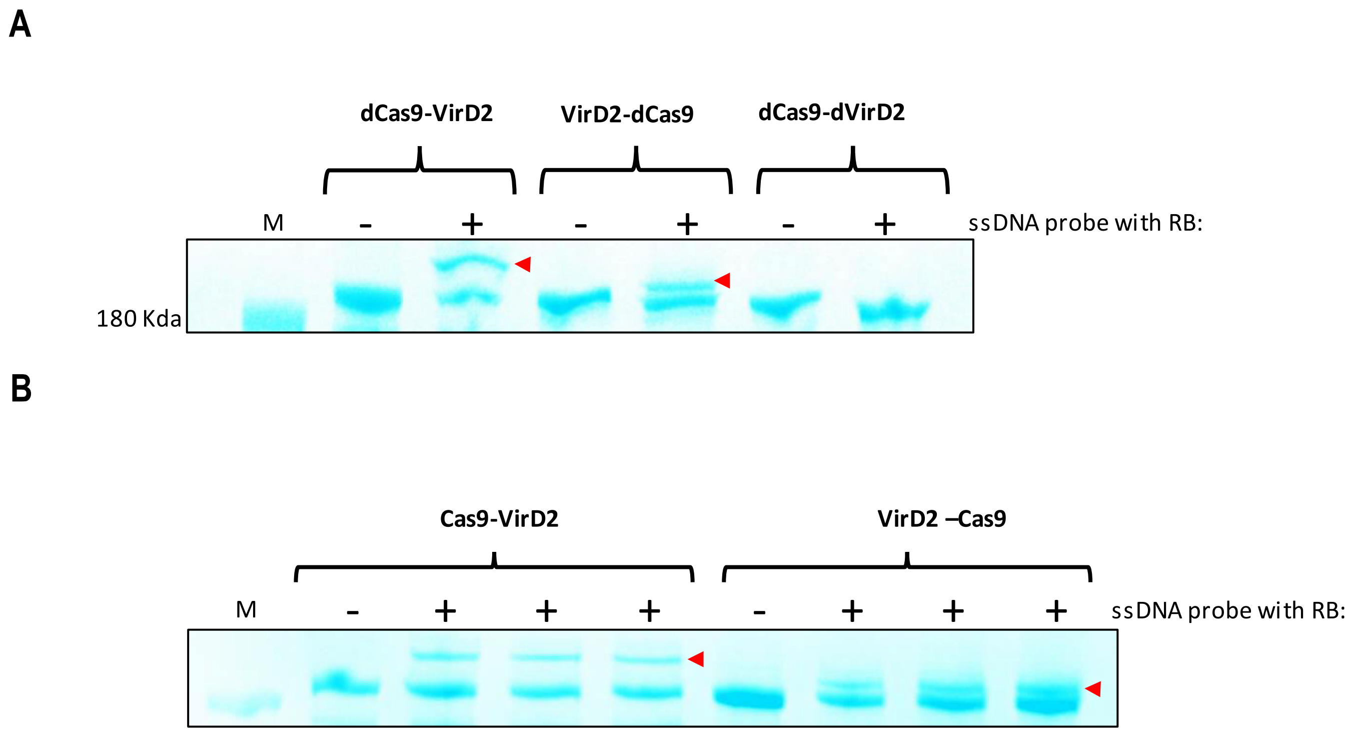

Supplementary Figure 3. Covalent binding of VirD2 and ssDNA. ssDNA strand (64 bp), harboring the T-DNA right border sequence was incubated with fusion proteins in the presence of Mg^{2+}. VirD2-Cas9, Cas9-VirD2, VirD2-dCas9, dCas9-VirD2 bound to biotin labeled probe were detected by Coomassie staining. RB containing probe did not bind dCas9-dVirD2. Red arrow head indicated the ssDNA - fusion protein complex.

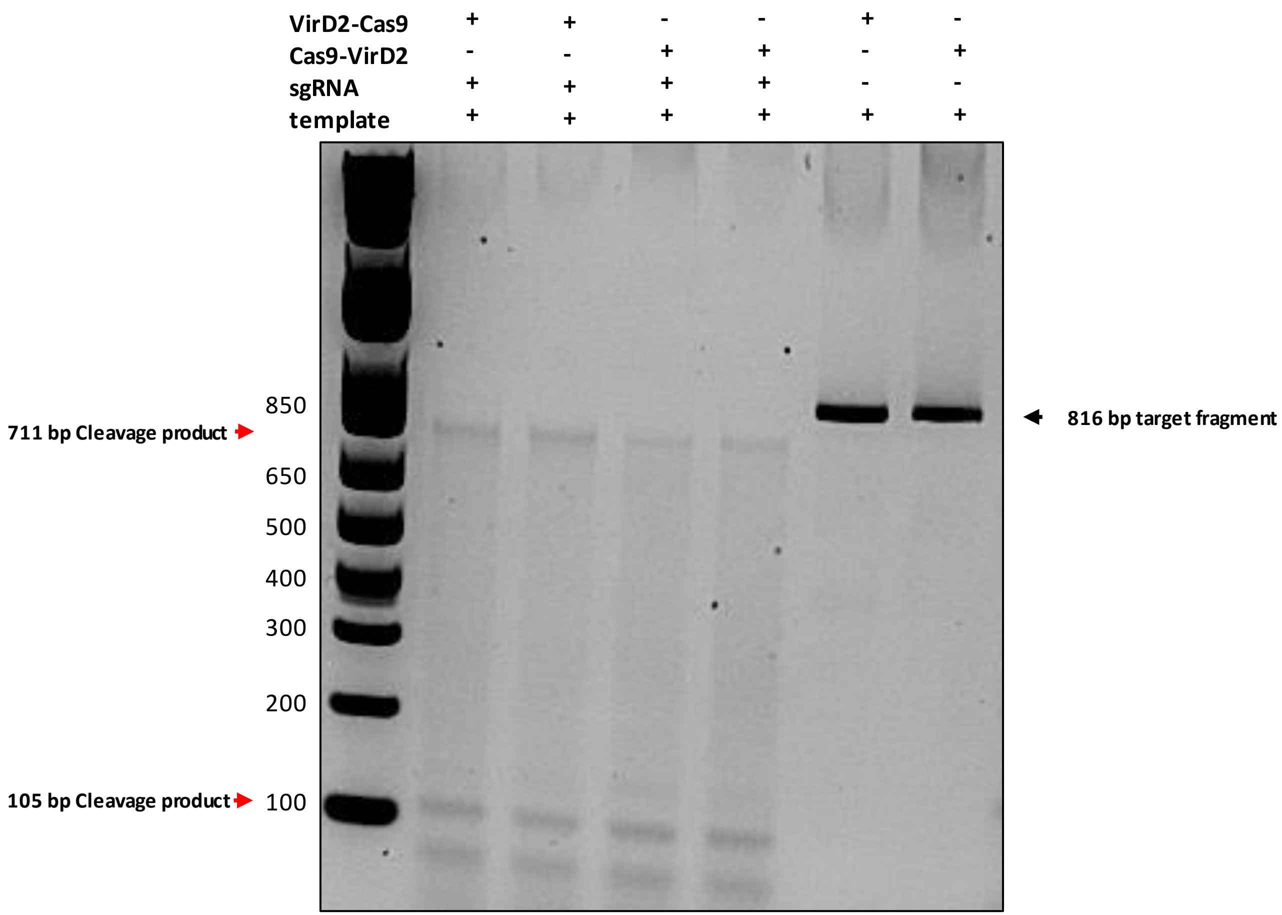

Supplementary Figure 4. Confirmation of VirD2-Cas9 and Cas9-VirD2 nuclease activity. N-gene target DNA was incubated with fusion proteins in the presence sgRNA and Mg^{2+}. Samples without sgRNA were used as controls. Arrow heads, indicate the respective DNA fragments.

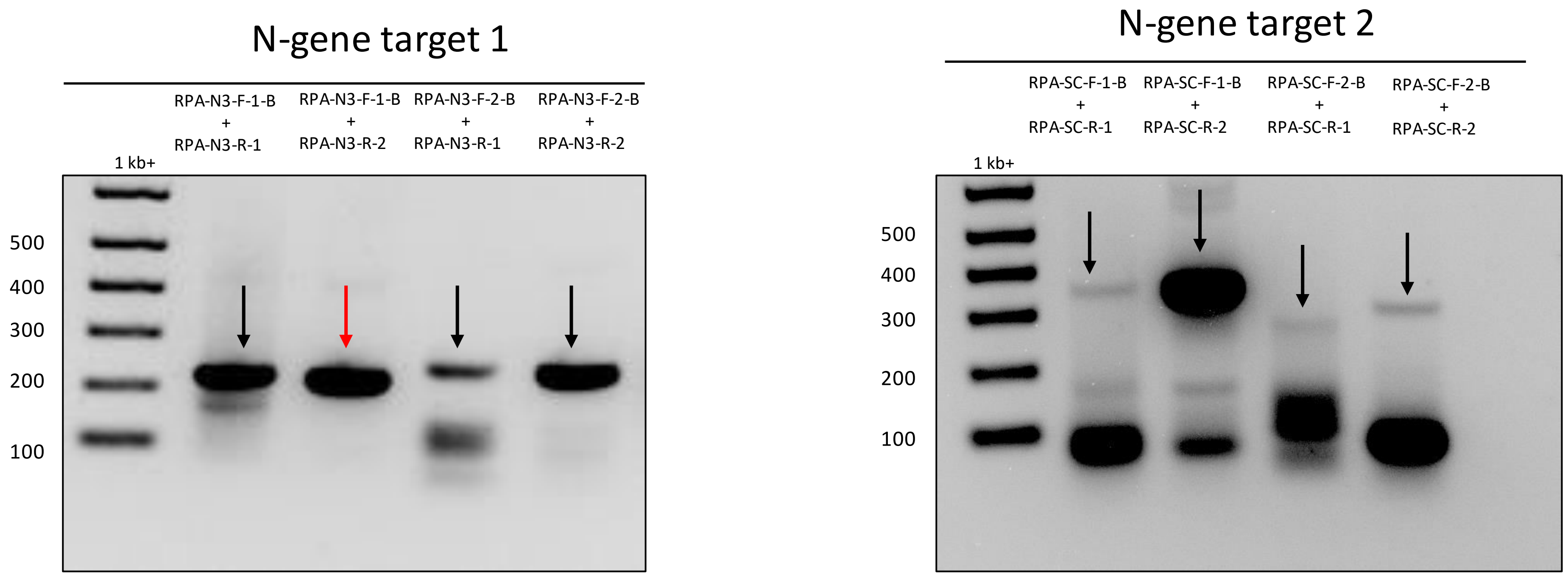

Supplementary Figure 5. Selection of the optimal RT-RPA primer set. N-gene target DNA was amplified by RT-RPA kit using manufacturer's instructions. Black arrow heads indicate the expected amplicons. Red arrow head, indicates the selected primer set. Primer set was selected based on the absence of non-specific amplicons and primer dimers.

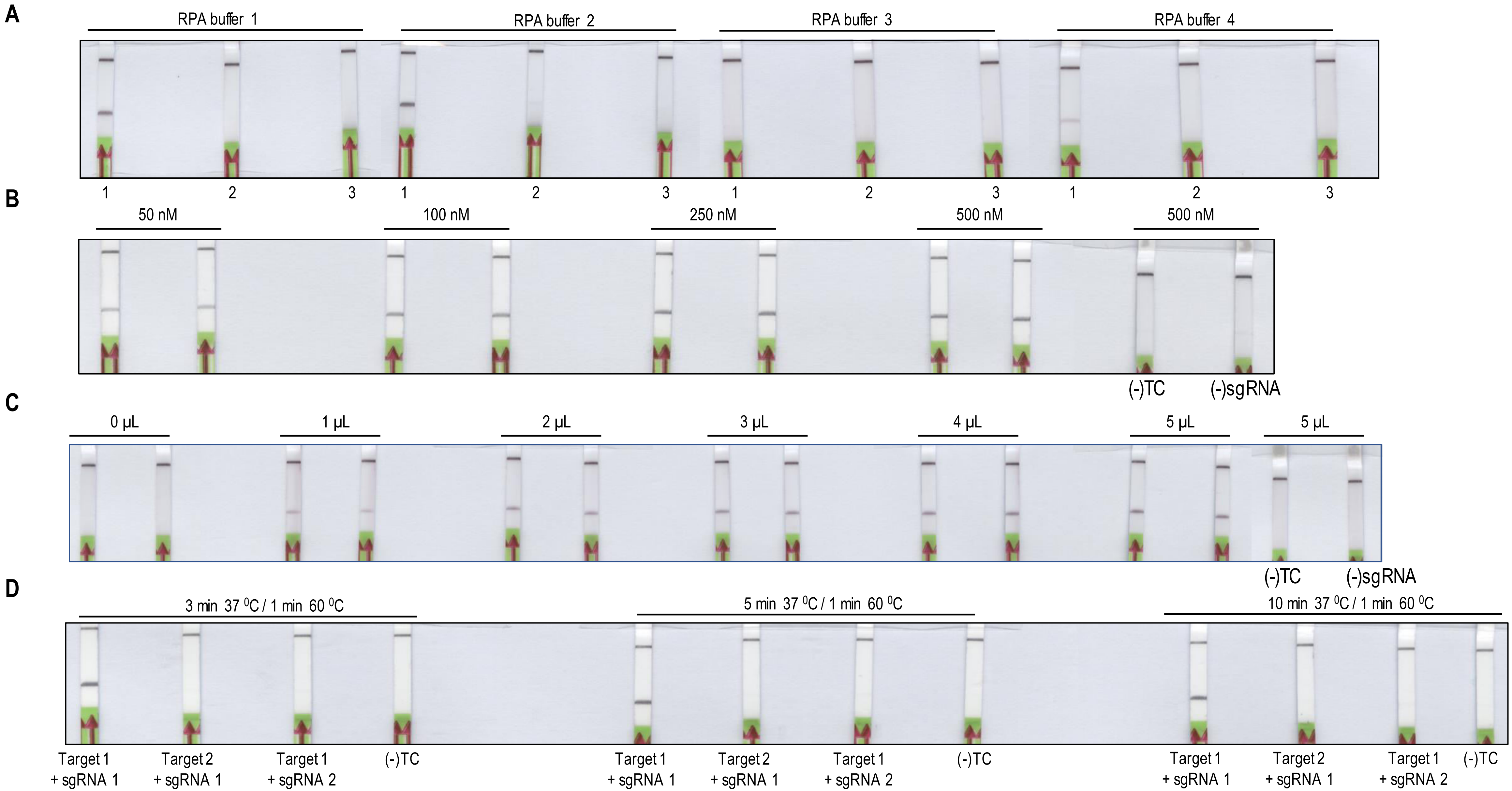

Supplementary Figure 6. A. Selection of the optimal reaction buffer. 1, specific target sample. 2, non-specific target sample. 3, non-specific sgRNA. **B.** Selection of the optimal reporter complex concentration. Protien:sgRNA:FAM-probe were mixed in 1:1:1 ratio in 50, 100, 250, 500 nM concentration in a 25 µL reaction volume. **C.** Selection of the optimal RT-RPA product volume for detection. Different volumes (0, 1, 2, 3, 4, 5 µL) of the RT-RPA product were added to the preassembled reporter complex. No template and no sgRNA were used as negative controls. **D.** Selection of the optimal duration for detection. RT-RPA product was incubated with preassembled reporter complex for different time periods. Incubation at 37 ^{0}C for 10 minutes followed by 1 minute at 60 ^{0}C results in robust, rapid and consistent appearance of the bands at the test line.

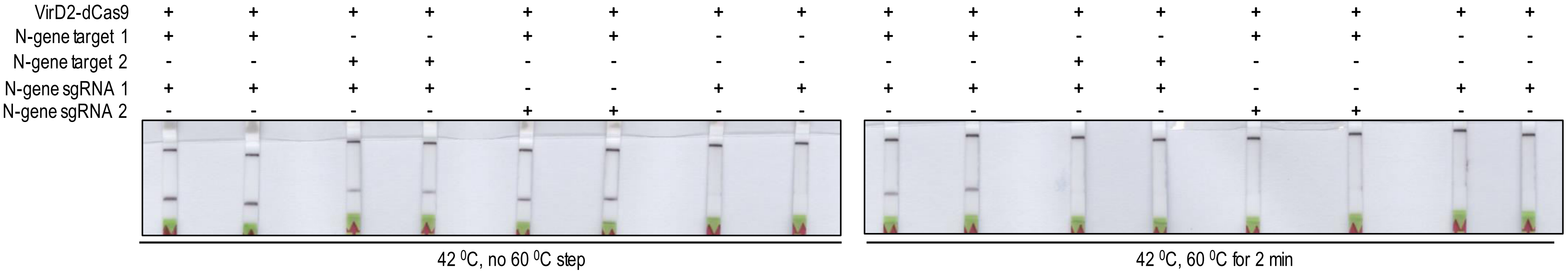

Supplementary Figure 7. Evaluation of the Vigilante at 42 ^{0}C. All steps, the RT-RPA reaction, reporter complex assembly and detection reaction were carried out at 42 ^{0}C. N-gene specific target and specific sgRNA were used as positive and no template samples was used as negative control.

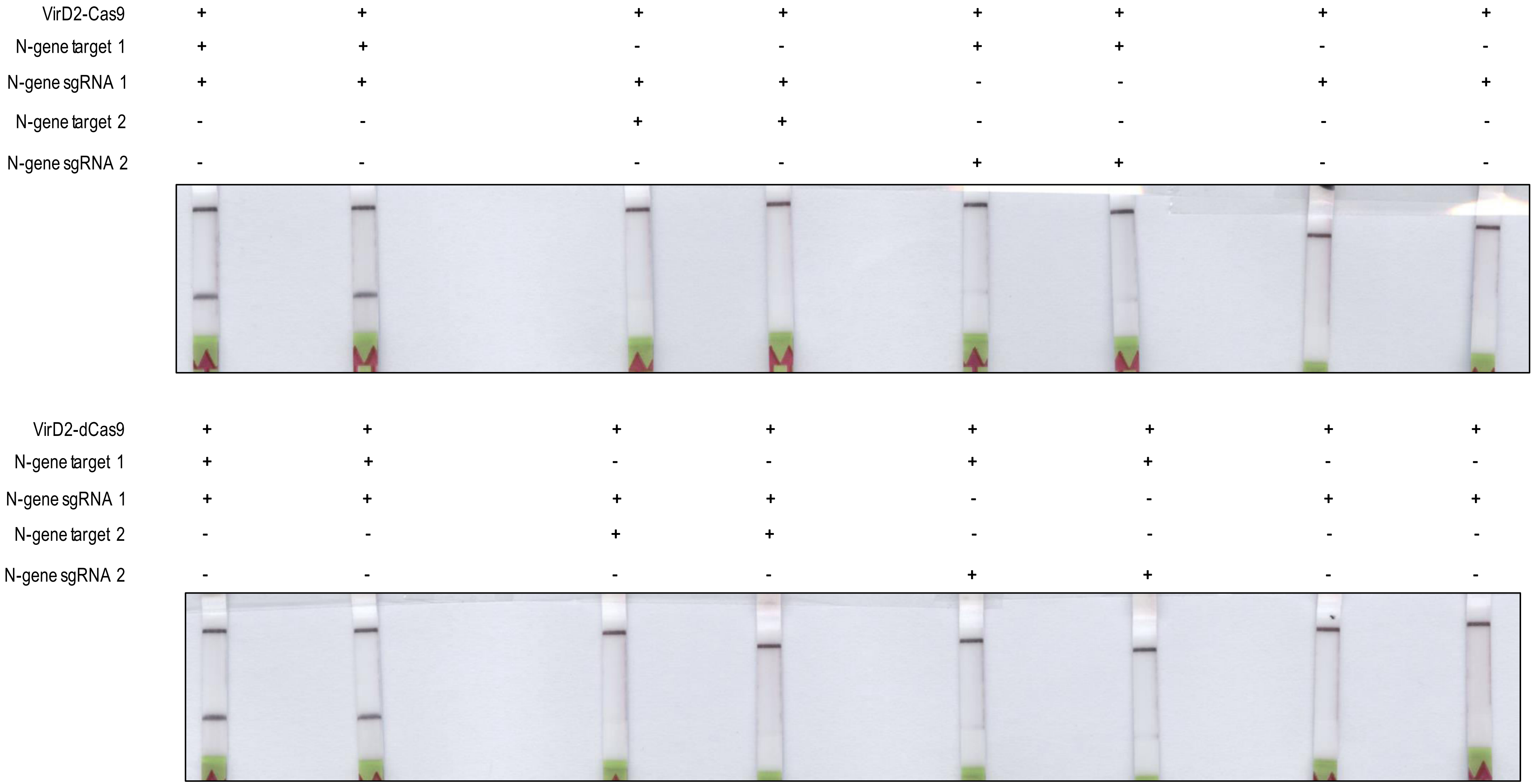

Supplementary Figure 8. Comparison of VirD2-Cas9 and VirD2-dCas9. SARS-CoV-2 RT-RPA amplified N-gene product was detected with the Vigilante platform. N-gene target 1, N-gene specific target; N-gene target 2, N-gene non-specific target; N-gene sgRNA 1, specific sgRNA; N-gene sgRNA 2, non-specific sgRNA

Replicates of the LoD experiment conducted in Figure 3A

Replicate 2

0	0	50	125	375	500	2500	5000	Copies per reaction
0	0	1	2.5	7.5	10	50	100	Copies per microliter

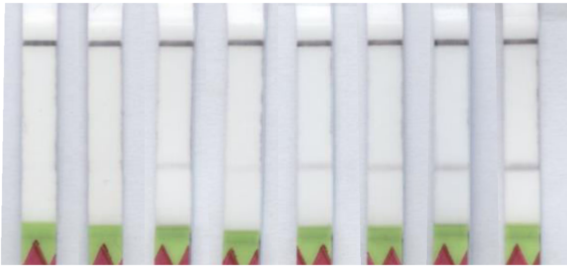

Replicate 3

0	0	50	125	375	500	2500	5000	Copies per reaction
0	0	1	2.5	7.5	10	50	100	Copies per microliter

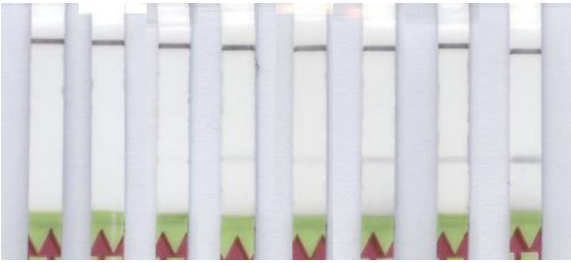

Supplementary Figure 9. Replicates of LoD experiment conducted in Figure 3A

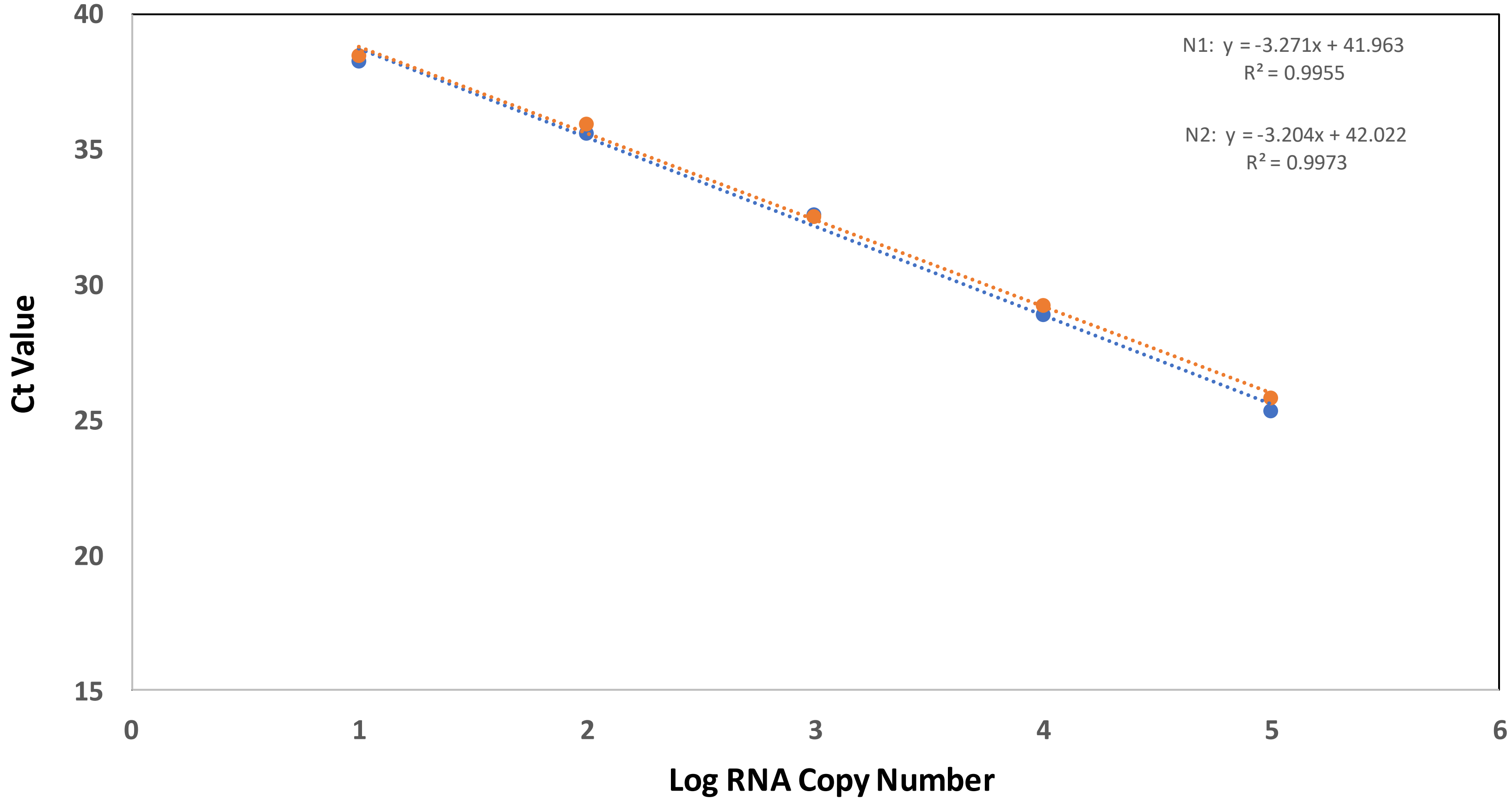

Supplementary Figure 10. Copy number determination by RT-qPCR for Ct value relevance in clinical samples. Synthetic SARS-CoV-2 RNA template was used to determine the LOD by RT-qPCR using One-step RT-qPCR kit (Invitrogen). Ct value was determined using two independent sets primer sets, N1 and N2. Blank sample (no template) was used as negative control.

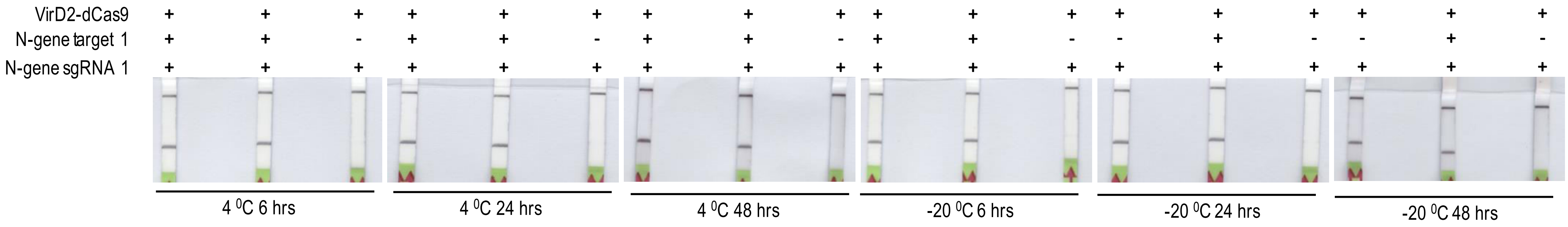

Supplementary Figure 11. Reporter complex stability. Protein:sgRNA:FAM-probe were mixed in 1:1:1 ratio in 250, nM concentration and incubated at 37 ^{0}C for 60 minutes. The reporter complex was stored at different temperatures for different periods of time. N-gene specific target and specific sgRNA were used as positive and no template samples was used as negative control.

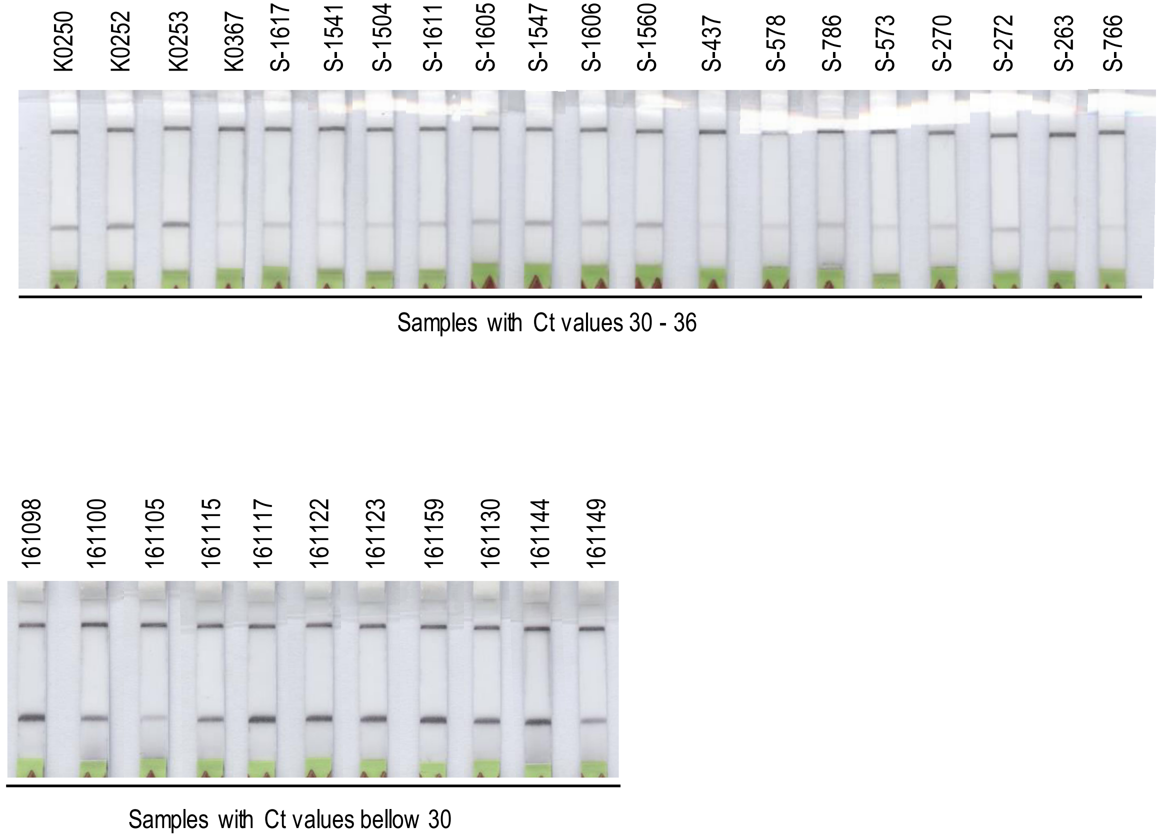

Supplementary Figure 12. Additional evaluated clinical samples and their original scan.

Sample designation	Ct value (RT-qPCR)
155418	21
155378	34
155381	21
155383	21
155384	14
155385	22
155388	22
155389	23
155369	16
155397	18
155398	23
155902	15
155905	20
155908	17
155916	22
155960	15
155974	26
156852	21
161058	17
161063	16.5
S1504	30.75
S1611	30.77
S1605	31.12
S1547	31.45
S1606	31.52
S1560	31.57
NID-1	>38
NID-2	>38
NID-3	>38
NID-4	>38

Sample	Ct value
K0250	34.64
K0252	36.06
K0253	35.57
K0367	35.16
S-1617	30.00
S-1541	30.55
S-1504	30.75
S-1611	30.77
S-1605	31.12
S-1547	31.45
S-1606	31.52
S-1560	31.57
S-437	32.47
S-578	33.25
S-786	32.88
S-573	33.25
S-270	33.80
S-272	32.70
S-263	33.29
S-766	31.99

Sample	Ct value
161098	29
161100	20
161105	23
161115	16
161117	15
161122	19
161123	22
161159	21
161130	18
161144	23
161149	19

Supplementary table 1. Designation and the Ct value of the clinical samples used in this study. RT-qPCR was performed on Trizol based isolated RNA.

Name	Sequence	Experimental description
virD2 reporter-FAM-S	5'-/gtttacccgccaatatatcctgtcaTTTTT/56-FAM/ -3'	Short, FAM-labeled probe for VirD2
virD2 reporter-FAM-L	5'-/gtttacccgccaatatatcctgtcaTTTTTTTTTT/56-FAM/-3'	Long, FAM-labeled probe for VirD2
virD2 reporter-B-L	5'-/gtttacccgccaatatatcctgtcaTTTTTTTTTT/biotin/-3'	Long, biotin-labeled probe for VirD2 used for Western-blot analysis of VirD2 nuclease- and binding activities
PCR-F1-N3	5'/biotin/CCGAAGAGCTACCAGACGAATTC/3'	Forward PCR primer used for proof-of-concept assay
PCR-R2-N3	5'/ TGTAGCACGATTGCAGCATTG/3'	Reverse PCR primer used for proof-of-concept assay
gBlock-N3-sgRNA-1	5'/GTCTCAGGCATAATACGACTCACTATAGGccagaagctggacttccctaGTTTTAGAGCTAGAAATAGCAAGTTAAAATAAGGCTAGTCCGTTATCAACTTGAAAAAGTGGCACCGAGTCGGTGC/3'	gBlock template used for IVT of sgRNA targeting both the PCR and RT-RPA generated amplicons
RPA-STOPCovid-F-1-B	5'/biotin/actaagaaatctgctgctgaggcttctaag	Used for primer screening
RPA-STOPCovid-R-1	5'/tgcgtcaatatgcttattcagcaaaatgac	Used for primer screening
RPA-STOPCovid-F-2-B	5'/biotin/gtactgccactaaagcatacaatgtaacac	Used for primer screening
RPA-STOPCovid-R-2	5'/gttttgtatgcgtcaatatgcttattcagc	Used for primer screening
RPA-N3-F-1-B	5'/biotin/agctaccagacgaattcgtggtggtgacgg	Selected RT-RPA fwd primer
RPA-N3-R-1	5'/ttgtagcacgattgcagcattgttagcagg	Used for primer screening
RPA-N3-F-2-B	5'/biotin/aattggctactaccgaagagctaccagacg	Used for primer screening
RPA-N3-R-2	5'/acgattgcagcattgttagcaggattgcgg	Selected RT-RPA rev primer
pstv-cnt-RB-ssDNA	cagatcaaTTCTCTTAGGTTTACCCGCCAATATATCCTGTCAAACACTGATAGTTTtcacagtagGGTG	Covalent binding confirmation of VirD2.dCas9 fusions

Sequences of 3 different mutant proteins

>3_SpdCas9-dVirD2-Mutated

Ccaccctgaattgactctcttccgggcgctatcatgccataccgcgaaaggttttgcgccattcgatggtgtccgggatctcgacgctctcccttatgcgactcctgcattaggaagcagcccagtagtaggttgag
gccgttgagcaccgccgccgcaaggaatggtgcatgcaaggagatggcgcccaacagtcccccggccacggggcctgccaccatacccacgccgaaacaagcgctcatgagcccgaagtggcgagcccgatc
ttccccatcggtgatgtcggcgatataggcgccagcaaccgcacctgtggcgccggtgatgccggccacgatgcgtccggcgtagaggatcgagatctcgatcccgcgaaattaatacgactcactatagggga
attgtgagcggataacaattcccctctagaaataattttgtttaactttaagaaggagatataccatgggcagcagccatcatcatcatcatcacagcagcggcctggtgccgcgcggcagccatatggctagcat
gactggtggacagcaaatgggtcgcggatccgaattcgagctccgtcgacaagcttgcggccgcatggacaagaagtacagcatcggcctggCTatcggtaccaacagcgtgggctgggccgtgatcaccgac
gagtacaaggtgcccagcaagaagttcaaggtgctgggcaacaccgaccgccacagcatcaagaagaacctgatcggcgccctgctgttcgacagcggcgagaccgccgaggccacccgcctgaagcgcacc
gcccgccgccgctacacccgccgcaagaaccgcatctgctacctgcaggagatcttcagcaacgagatggccaaggtggacgacagcttcttccaccgcctggaggagagcttcctggtggaggaggacaaga
agcacgagcgccaccccatcttcggcaacatcgtggacgaggtggcctaccacgagaagtaccccaccatctaccacctgcgcaagaagctggtggacagcaccgacaaggccgacctgcgcctgatctacct
ggccctggcccacatgatcaagttccgcggccacttcctgatcgagggcgacctgaaccccgacaacagcgacgtggacaagctgttcatccagctggtgcagacctacaaccagctgttcgaggagaacccca
tcaacgccagcggcgtggacgccaaggccatcctgagcgcccgcctgagcaagagccgccgcctggagaacctgatcgcccagctgcccggcgagaagaagaacggcctgttcggcaacctgatcgccctga
gcctgggcctgacccccaacttcaagagcaacttcgacctggccgaggacgccaagctgcagctgagcaaggacacctacgacgacgacctggacaacctgctggcccagatcggcgaccagtacgccgacct
gttcctggccgccaagaacctgagcgacgccatcctgctgagcgacatcctgcgcgtgaacaccgagatcaccaaggcccccctgagcgccagcatgatcaagcgctacgacgagcaccaccaggacctgacc
ctgctgaaggccctggtgcgccagcagctgcccgagaagtacaaggagatcttcttcgaccagagcaagaacggctacgccggctacatcgacggcggcgccagccaggaggagttctacaagttcatcaagc
ccatcctggagaagatggacggcaccgaggagctgctggtgaagctgaaccgcgaggacctgctgcgcaagcagcgcaccttcgacaacggcagcatcccccaccagatccacctgggcgagctgcacgccat
cctgcgccgccaggaggacttctaccccttcctgaaggacaaccgcgagaagatcgagaagatcctgaccttccgcatcccctactacgtgggccccctggcccgcggcaacagccgcttcgcctggatgacccg
caagagcgaggagaccatcaccccctggaacttcgaggaggtggtggacaagggcgccagcgcccagagcttcatcgagcgcatgaccaacttcgacaagaacctgcccaacgagaaggtgctgcccaagca
cagcctgctgtacgagtacttcaccgtgtacaacgagctgaccaaggtgaagtacgtgaccgagggcatgcgcaagcccgccttcctgagcggcgagcagaagaaggccatcgtggacctgctgttcaagacca
accgcaaggtgaccgtgaagcagctgaaggaggactacttcaagaagatcgagtgcttcgacagcgtggagatcagcggcgtggaggaccgcttcaacgccagcctgggcacctaccacgacctgctgaagat
catcaaggacaaggacttcctggacaacgaggagaacgaggacatcctggaggacatcgtgctgaccctgaccctgttcgaggaccgcgagatgatcgaggagcgcctgaagacctacgcccacctgttcgac
gacaaggtgatgaagcagctgaagcgccgccgctacaccggctggggccgcctgagccgcaagcttatcaacggcatccgcgacaagcagagcggcaagaccatcctggacttcctgaagagcgacggcttc
gccaaccgcaacttcatgcagctgatccacgacgacagcctgaccttcaaggaggacatccagaaggcccaggtgagcggccagggcgacagcctgcacgagcacatcgccaacctggccggcagccccgcc
atcaagaagggcatcctgcagaccgtgaaggtggtggacgagctggtgaaggtgatgggccgccacaagcccgagaacatcgtgatcgagatggcccgcgagaaccagaccacccagaagggccagaaga
acagccgcgagcgcatgaagcgcatcgaggagggcatcaaggagctgggcagccagatcctgaaggagcaccccgtggagaacacccagctgcagaacgagaagctgtacctgtactacctgcagaacggc
cgcgacatgtacgtggaccaggagctggacatcaaccgcctgagcgactacgacgtggacGCcatcgtgccccagagcttcctgaaggacgacagcatcgacaacaaggtgctgacccgcagcgacaagaa
ccgcggcaagagcgacaacgtgcccagcgaggaggtggtgaagaagatgaagaactactggcgccagctgctgaacgccaagctgatcacccagcgcaagttcgacaacctgaccaaggccgagcgcggcg
gcctgagcgagctggacaaggccggcttcatcaagcgccagctggtggagacccgccagatcaccaagcacgtggcccagatcctggacagccgcatgaacaccaagtacgacgagaacgacaagctgatcc
gcgaggtgaaggtgatcaccctgaagagcaagctggtgagcgacttccgcaaggacttccagttctacaaggtgcgcgagatcaacaactaccaccacgcccacgacgcctacctgaacgccgtggtgggcac

cgccctgatcaagaagtaccccaagctggagagcgagttcgtgtacggcgactacaaggtgtacgacgtgcgcaagatgatcgccaagagcgagcaggagatcggcaaggccaccgccaagtacttcttctac
agcaacatcatgaacttcttcaagaccgagatcaccctggccaacggcgagatccgcaagcgcccccctgatcgagaccaacggcgagaccggcgagatcgtgtgggacaagggccgcgacttcgccaccgtgc
gcaaggtgctgagcatgccccaggtgaacatcgtgaagaagaccgaggtgcagaccggcggcttcagcaaggagagcatcctgcccaagcgcaacagcgacaagctgatcgcccgcaagaaggactgggac
cccaagaagtacggcggcttcgacagccccaccgtggcctacagcgtgctggtggtggccaaggtggagaagggcaagagcaagaagctgaagagcgtgaaggagctgctgggcatcaccatcatggagcgc
agcagcttcgagaagaaccccatcgacttcctggaggccaagggctacaaggaggtgaagaaggacctgatcatcaagctgcccaagtacagcctgttcgagctggagaacggccgcaagcgcatgctggcc
agcgccggcgagctgcagaagggcaacgagctggccctgcccagcaagtacgtgaacttcctgtacctggccagccactacgagaagctgaagggcagccccgaggacaacgagcagaagcagctgttcgtg
gagcagcacaagcactacctggacgagatcatcgagcagatcagcgagttcagcaagcgcgtgatcctggccgacgccaacctggacaaggtgctgagcgcctacaacaagcaccgcgacaagcccatccgc
gagcaggccgagaacatcatccacctgttcaccctgaccaacctgggcgccccgccgccttcaagtacttcgacaccaccatcgaccgcaagcgctacaccagcaccaaggaggtgctggacgccaccctgat
ccaccagagcatcaccggtctgtacgagacccgcatcgacctgagccagctgggcggcgacggcggctccggacctccaaagaaaaagagaaaagtataccccctacgacgtgcccgactacgcctgtggagg
cggtggatctggcggtggcggaagtgggggaggtggaagtggtaccatgcccgatcgagctcaagttatcattcgcattgtgccgggaggtggcaccaagacccttcaacaaattatcaatcagttggagGCCc
tatcccggaagggcaggctggagctgcagcgttcagcccgacatctcgatattcccctgccaccggatcaaatccacgaacttgcccgaagctgggttcaagagactggaacttatgacgaaagtcagccagac
gaggaaaggcaacaggagttgaccacccatattattgtaagcttccccgccggtacaagccaggtagcggcttatgcggcgagccgggagtgggcagccgagatgtttgggtcaggcgcaggggggggccgat
acaactatcttacggccttccacatcgatcgcgaccacccacatctgcatgtcgtcgtcaatcggcgcgaacttttaggacacggctggctgaagatatctcggcgccatccccaactgaattacgacgccctgcg
cataaagatggccgagatttcacttcgtcatggcattgccctcgatgcgagccgacgagcagaacgtggcatcaccgagcggccgatcacttatgcccaatatcggcgccttgagcgggagcaggctcgccaaa
tccgtttcgaagacgcggatttggaacagtcgtcgccgcaaggagatcatccagaattcagccaacctttcgatacatccccatttgaagcatccgcgggcggaccggaggacatgcctcggcccaacaatcggc
agaatgagtcgcaagttcatctccaggagccagctggtgtcagcaacgaagccggtgtccttgtgcgggttgcattggagacggagcgccttgctcaaccattcgtttccgaaaccattctcgcggacgacatagg
gagcggctcttcgcgtgttgccgagggccgtgtggagagcgcaaaccgcactcccgatattcctcgcgcagcaactgaagctgccacgcacacgacacacgaccggcagcggcgtgcaaagcgtcctcatgatg
acgacggagggccgagtggagcaaaacgtgtgacattggaaggcatcgcggttgggccccaggcgaacgccggcgaacaggatggcagtagtggccccttagtacggcaagctggaacgtctcggccatctc
caccgacggccacgacgcgggccagcaccgcaaccgattcattgtctgctacagcccacctccagcaacggagaggtgtcctttcaaagcgtccgcgtgaagatgatgatggagaaccgagtgaacgcaaacg
cgagagagatgagcgcagcaaggacgggcgtgggggaaataggagataataagcggccgcactcgagcaccaccaccaccaccactgagatccggctgctaacaaagcccgaaaggaagctgagttggctg
ctgccaccgctgagcaataactagcataaccccttggggcctctaaacgggtcttgaggggttttttgctgaaaggaggaactatatccggattggcgaatgggacgcgccctgtagcggcgcattaagcgcggc
gggtgtggtggttacgcgcagcgtgaccgctacacttgccagcgccctagcgcccgctcctttcgctttcttcccttcctttctcgccacgttcgccggctttccccgtcaagctctaaatcgggggctccctttagggt
tccgatttagtgctttacggcacctcgaccccaaaaaacttgattagggtgatggttcacgtagtgggccatcgccctgatagacggtttttcgccctttgacgttggagtccacgttctttaatagtggactcttgttc
caaactggaacaacactcaaccctatctcggtctattcttttgatttataagggattttgccgatttcggcctattggttaaaaaatgagctgatttaacaaaaatttaacgcgaattttaacaaaatattaacgctta
caatttaggtggcacttttcggggaaatgtgcgcggaacccctatttgtttatttttctaaatacattcaaatatgtatccgctcatgaattaattcttagaaaaactcatcgagcatcaaatgaaactgcaatttatt
catatcaggattatcaataccatatttttgaaaaagccgtttctgtaatgaaggagaaaactcaccgaggcagttccataggatggcaagatcctggtatcggtctgcgattccgactcgtccaacatcaatacaa
cctattaatttcccctcgtcaaaaataaggttatcaagtgagaaatcaccatgagtgacgactgaatccggtgagaatggcaaaagtttatgcatttctttccagacttgttcaacaggccagccattacgctcgtc
atcaaaatcactcgcatcaaccaaaccgttattcattcgtgattgcgcctgagcgagacgaaatacgcgatcgctgttaaaaggacaattacaaacaggaatcgaatgcaaccggcgcaggaacactgccagc
gcatcaacaatattttcacctgaatcaggatattcttctaatacctggaatgctgttttcccggggatcgcagtggtgagtaaccatgcatcatcaggagtacggataaaatgcttgatggtcggaagaggcataa
attccgtcagccagtttagtctgaccatctcatctgtaacatcattggcaacgctacctttgccatgtttcagaaacaactctggcgcatcgggcttcccatacaatcgatagattgtcgcacctgattgcccgacatt
atcgcgagcccatttatacccatataaatcagcatccatgttggaatttaatcgcggcctagagcaagacgtttcccgttgaatatggctcataacaccccttgtattactgtttatgtaagcagacagttttattgtt
catgaccaaaatcccttaacgtgagttttcgttccactgagcgtcagaccccgtagaaaagatcaaaggatcttcttgagatcctttttttctgcgcgtaatctgctgcttgcaaacaaaaaaaccaccgctaccag
cggtggtttgtttgccggatcaagagccaccaactctttttccgaaggtaactggcttcagcagagcgcagataccaaatactgtccttctagtgtagccgtagttaggccaccacttcaagaactctgtagcaccg
cctacatacctcgctctgctaatcctgttaccagtggctgctgccagtggcgataagtcgtgtcttaccgggttggactcaagacgatagttaccggataaggcgcagcggtcgggctgaacggggggttcgtgca
cacagcccagcttggagcgaacgacctacaccgaactgagatacctacagcgtgagctatgagaaagcgccacgcttcccgaagggagaaaggcggacaggtatccggtaagcggcagggtcggaacagga
gagcgcacgagggagcttccagggggaaacgcctggtatctttatagtcctgtcgggtttcgccacctctgacttgagcgtcgatttttgtgatgctcgtcaggggggcggagcctatggaaaaacgccagcaacg
cggcctttttacggttcctggccttttgctggccttttgctcacatgttctttcctgcgttatcccctgattctgtggataaccgtattaccgcctttgagtgagctgataccgctcgccgcagccgaacgaccgagcgca
gcgagtcagtgagcgaggaagcggaagagcgcctgatgcggtattttctccttacgcatctgtgcggtatttcacaccgcaatggtgcactctcagtacaatctgctctgatgccgcatagttaagccagtatacac
tccgctatcgctacgtgactgggtcatggctgcgccccgacacccgccaacacccgctgacgcgccctgacgggcttgtctgctcccggcatccgcttacagacaagctgtgaccgtctccgggagctgcatgtgtc
agaggttttcaccgtcatcaccgaaacgcgcgaggcagctgcggtaaagctcatcagcgtggtcgtgaagcgattcacagatgtctgcctgttcatccgcgtccagctcgttgagtttctccagaagcgttaatgtc
tggcttctgataaagcgggccatgttaagggcggtttttttcctgtttggtcactgatgcctccgtgtaagggggatttctgttcatgggggtaatgataccgatgaaacgagagaggatgctcacgatacgggttact
gatgatgaacatgcccggttactggaacgttgtgagggtaaacaactggcggtatggatgcggcgggaccagagaaaaatcactcagggtcaatgccagcgcttcgttaatacagatgtaggtgttccacaggg
tagccagcagcatcctgcgatgcagatccggaacataatggtgcagggcgctgacttccgcgtttccagactttacgaaacacggaaaccgaagaccattcatgttgttgctcaggtcgcagacgttttgcagca
gcagtcgcttcacgttcgctcgcgtatcggtgattcattctgctaaccagtaaggcaaccccgccagcctagccgggtcctcaacgacaggagcacgatcatgcgcacccgtggggccgccatgccggcgataat
ggcctgcttctcgccgaaacgtttggtggcgggaccagtgacgaaggcttgagcgagggcgtgcaagattccgaataccgcaagcgacaggccgatcatcgtcgcgctccagcgaaagcggtcctcgccgaaa
atgacccagagcgctgccggcacctgtcctacgagttgcatgataaagaagacagtcataagtgcggcgacgatagtcatgccccgcgcccaccggaaggagctgactgggttgaaggctctcaagggcatcg
gtcgagatcccggtgcctaatgagtgagctaacttacattaattgcgttgcgctcactgcccgctttccagtcgggaaacctgtcgtgccagctgcattaatgaatcggccaacgcgcggggagaggcggtttgcg
tattgggcgccagggtggtttttcttttcaccagtgagacgggcaacagctgattgcccttcaccgcctggccctgagagagttgcagcaagcggtccacgctggtttgccccagcaggcgaaaatcctgtttgatg
gtggttaacggcgggatataacatgagctgtcttcggtatcgtcgtatcccactaccgagatatccgcaccaacgcgcagcccggactcggtaatggcgcgcattgcgcccagcgccatctgatcgttggcaacca
gcatcgcagtgggaacgatgccctcattcagcatttgcatggtttgttgaaaaccggacatggcactccagtcgccttcccgttccgctatcggctgaatttgattgcgagtgagatatttatgccagccagccaga
cgcagacgcgccgagacagaacttaatgggcccgctaacagcgcgatttgctggtgacccaatgcgaccagatgctccacgcccagtcgcgtaccgtcttcatgggagaaaataatactgttgatgggtgtctgg
tcagagacatcaagaaataacgccggaacattagtgcaggcagcttccacagcaatggcatcctggtcatccagcggatagttaatgatcagcccactgacgcgttgcgcgagaagattgtgcaccgccgcttta
caggcttcgacgccgcttcgttctaccatcgacaccaccacgctggcacccagttgatcggcgcgagatttaatcgccgcgacaatttgcgacggcgcgtgcagggccagactggaggtggcaacgccaatcag
caacgactgtttgcccgccagttgttgtgccacgcggttgggaatgtaattcagctccgccatcgccgcttccactttttcccgcgttttcgcagaaacgtggctggcctggttcaccacgcgggaaacggtctgata
agagacaccggcatactctgcgacatcgtataacgttactggtttcacattca

>5_VirD2-SpdCas9-Mutated

tttaacgcgaattttaacaaaatattaacgcttacaatttaggtggcacttttcggggaaatgtgcgcggaacccctatttgtttatttttctaaatacattcaaatatgtatccgctcatgaattaattcttagaaaaa
ctcatcgagcatcaaatgaaactgcaatttattcatatcaggattatcaataccatatttttgaaaaagccgtttctgtaatgaaggagaaaactcaccgaggcagttccataggatggcaagatcctggtatcggt
ctgcgattccgactcgtccaacatcaatacaacctattaatttcccctcgtcaaaaataaggttatcaagtgagaaatcaccatgagtgacgactgaatccggtgagaatggcaaaagtttatgcatttctttccag
acttgttcaacaggccagccattacgctcgtcatcaaaatcactcgcatcaaccaaaccgttattcattcgtgattgcgcctgagcgagacgaaatacgcgatcgctgttaaaaggacaattacaaacaggaatc
gaatgcaaccggcgcaggaacactgccagcgcatcaacaatattttcacctgaatcaggatattcttctaatacctggaatgctgttttcccggggatcgcagtggtgagtaaccatgcatcatcaggagtacgga
taaaatgcttgatggtcggaagaggcataaattccgtcagccagtttagtctgaccatctcatctgtaacatcattggcaacgctacctttgccatgtttcagaaacaactctggcgcatcgggcttcccatacaatc
gatagattgtcgcacctgattgcccgacattatcgcgagcccatttatacccatataaatcagcatccatgttggaatttaatcgcggcctagagcaagacgtttcccgttgaatatggctcataacaccccttgtat
tactgtttatgtaagcagacagttttattgttcatgaccaaaatcccttaacgtgagttttcgttccactgagcgtcagaccccgtagaaaagatcaaaggatcttcttgagatcctttttttctgcgcgtaatctgctg

cttgcaaacaaaaaaaccaccgctaccagcggtggtttgtttgccggatcaagagccaccaactctttttccgaaggtaactggcttcagcagagcgcagataccaaatactgtccttctagtgtagccgtagtta
ggccaccacttcaagaactctgtagcaccgcctacatacctcgctctgctaatcctgttaccagtggctgctgccagtggcgataagtcgtgtcttaccgggttggactcaagacgatagttaccggataaggcgca
gcggtcgggctgaacggggggttcgtgcacacagcccagcttggagcgaacgacctacaccgaactgagatacctacagcgtgagctatgagaaagcgccacgcttcccgaagggagaaaggcggacaggta
tccggtaagcggcagggtcggaacaggagagcgcacgagggagcttccagggggaaacgcctggtatctttatagtcctgtcgggtttcgccacctctgacttgagcgtcgatttttgtgatgctcgtcaggggg
cggagcctatggaaaaacgccagcaacgcggcctttttacggttcctggccttttgctggccttttgctcacatgttctttcctgcgttatcccctgattctgtggataaccgtattaccgcctttgagtgagctgatac
cgctcgccgcagccgaacgaccgagcgcagcgagtcagtgagcgaggaagcggaagagcgcctgatgcggtattttctccttacgcatctgtgcggtatttcacaccgcaatggtgcactctcagtacaatctgc
tctgatgccgcatagttaagccagtatacactccgctatcgctacgtgactgggtcatggctgcgccccgacacccgccaacacccgctgacgcgccctgacgggcttgtctgctcccggcatccgcttacagaca
agctgtgaccgtctccgggagctgcatgtgtcagaggttttcaccgtcatcaccgaaacgcgcgaggcagctgcggtaaagctcatcagcgtggtcgtgaagcgattcacagatgtctgcctgttcatccgcgtcc
agctcgttgagtttctccagaagcgttaatgtctggcttctgataaagcgggccatgttaagggcggtttttcctgtttggtcactgatgcctccgtgtaagggggatttctgttcatgggggtaatgataccgatga
aacgagagaggatgctcacgatacgggttactgatgatgaacatgcccggttactggaacgttgtgagggtaaacaactggcggtatggatgcggcgggaccagagaaaaatcactcagggtcaatgccagcg
cttcgttaatacagatgtaggtgttccacagggtagccagcagcatcctgcgatgcagatccggaacataatggtgcagggcgctgacttccgcgtttccagactttacgaaacacggaaaccgaagaccattca
tgttgttgctcaggtcgcagacgttttgcagcagcagtcgcttcacgttcgctcgcgtatcggtgattcattctgctaaccagtaaggcaaccccgccagcctagccgggtcctcaacgacaggagcacgatcatgc
gcacccgtggggccgccatgccggcgataatggcctgcttctcgccgaaacgtttggtggcgggaccagtgacgaaggcttgagcgagggcgtgcaagattccgaataccgcaagcgacaggccgatcatcgtc
gcgctccagcgaaagcggtcctcgccgaaaatgacccagagcgctgccggcacctgtcctacgagttgcatgataaagaagacagtcataagtgcggcgacgatagtcatgccccgcgcccaccggaaggagc
tgactgggttgaaggctctcaagggcatcggtcgagatcccggtgcctaatgagtgagctaacttacattaattgcgttgcgctcactgcccgctttccagtcgggaaacctgtcgtgccagctgcattaatgaatc
ggccaacgcgcggggagaggcggtttgcgtattgggcgccagggtggtttttcttttcaccagtgagacgggcaacagctgattgcccttcaccgcctggccctgagagagttgcagcaagcggtccacgctggtt
tgccccagcaggcgaaaatcctgtttgatggtggttaacggcgggatataacatgagctgtcttcggtatcgtcgtatcccactaccgagatatccgcaccaacgcgcagcccggactcggtaatggcgcgcattg
cgcccagcgccatctgatcgttggcaaccagcatcgcagtgggaacgatgccctcattcagcatttgcatggtttgttgaaaaccggacatggcactccagtcgccttcccgttccgctatcggctgaatttgattgc
gagtgagatatttatgccagccagccagacgcagacgcgccgagacagaacttaatgggcccgctaacagcgcgatttgctggtgacccaatgcgaccagatgctccacgcccagtcgcgtaccgtcttcatgg
gagaaaataatactgttgatgggtgtctggtcagagacatcaagaaataacgccggaacattagtgcaggcagcttccacagcaatggcatcctggtcatccagcggatagttaatgatcagcccactgacgcg
ttgcgcgagaagattgtgcaccgccgctttacaggcttcgacgccgcttcgttctaccatcgacaccaccacgctggcacccagttgatcggcgcgagatttaatcgccgcgacaatttgcgacggcgcgtgcagg
gccagactggaggtggcaacgccaatcagcaacgactgtttgcccgccagttgttgtgccacgcggttgggaatgtaattcagctccgccatcgccgcttccactttttcccgcgttttcgcagaaacgtggctggc
ctggttcaccacgcgggaaacggtctgataagagacaccggcatactctgcgacatcgtataacgttactggtttcacattcaccaccctgaattgactctcttccgggcgctatcatgccataccgcgaaaggttt
tgcgccattcgatggtgtccgggatctcgacgctctcccttatgcgactcctgcattaggaagcagcccagtagtaggttgaggccgttgagcaccgccgccgcaaggaatggtgcatgcaaggagatggcgccc
aacagtcccccggccacggggcctgccaccatacccacgccgaaacaagcgctcatgagcccgaagtggcgagcccgatcttccccatcggtgatgtcggcgatataggcgccagcaaccgcacctgtggcgc
cggtgatgccggccacgatgcgtccggcgtagaggatcgagatctcgatcccgcgaaattaatacgactcactataggggaattgtgagcggataacaattcccctctagaaataattttgtttaactttaagaag
gagatataccatgggcagcagccatcatcatcatcatcacagcagcggcctggtgccgcgcggcagccatatggctagcatgactggtggacagcaaatgggtcgcggatccgaattcgagctccgtcgacaag
cttgcggccgcatgcccgatcgagctcaagttatcattcgcattgtgccgggaggtggcaccaagacccttcaacaaattatcaatcagttggagtatctatcccggaagggcaggctggagctgcagcgttcagc
ccgacatctcgatattcccctgccaccggatcaaatccacgaacttgcccgaagctgggttcaagagactggaacttatgacgaaagtcagccagacgaggaaaggcaacaggagttgaccacccatattattg
taagcttccccgccggtacaagccaggtagcggcttatgcggcgagccgggagtgggcagccgagatgtttgggtcaggcgcagggggggggccgatacaactatcttacggccttccacatcgatcgcgaccac
ccacatctgcatgtcgtcgtcaatcggcgcgaactttaggacacggctggctgaagatatctcggcgccatccccaactgaattacgacgccctgcgcataaagatggccgagatttcacttcgtcatggcattgc
cctcgatgcgagccgacgagcagaacgtggcatcaccgagcggccgatcacttatgcccaatatcggcgccttgagcgggagcaggctcgccaaatccgtttcgaagacgcggatttggaacagtcgtcgccgc
aaggagatcatccagaattcagccaacctttcgatacatccccatttgaagcatccgcgggcggaccggaggacatgcctcggcccaacaatcggcagaatgagtcgcaagttcatctccaggagccagctggt
gtcagcaacgaagccggtgtccttgtgcgggttgcattggagacggagcgccttgctcaaccattcgtttccgaaaccattctcgcggacgacatagggagcggctcttcgcgtgttgccgagggccgtgtggaga
gcgcaaaccgcactcccgatattcctcgcgcagcaactgaagctgccacgcacacgacacacgaccggcagcggcgtgcaaagcgtcctcatgatgacgacggagggccgagtggagcaaaacgtgtgacat
tggaaggcatcgcggttgggccccaggcgaacgccggcgaacaggatggcagtagtggccccttagtacggcaagctggaacgtctcggccatctccaccgacggccacgacgcgggccagcaccgcaaccg
attcattgtctgctacagcccacctccagcaacggagaggtgtcctttcaaagcgtccgcgtgaagatgatgatggagaaccgagtgaacgcaaacgcgagagagatgagcgcagcaaggacgggcgtgggg
gaaataggagaagatctggaggcggtggatctggcggtggcggaagtgggggaggtggaagtatggacaagaagtacagcatcggcctggCTatcggtaccaacagcgtgggctgggccgtgatcaccgac
gagtacaaggtgcccagcaagaagttcaaggtgctgggcaacaccgaccgccacagcatcaagaagaacctgatcggcgccctgctgttcgacagcggcgagaccgccgaggccacccgcctgaagcgcacc
gcccgccgccgctacacccgccgcaagaaccgcatctgctacctgcaggagatcttcagcaacgagatggccaaggtggacgacagcttcttccaccgcctggaggagagcttcctggtggaggaggacaaga
agcacgagcgccaccccatcttcggcaacatcgtggacgaggtggcctaccacgagaagtaccccaccatctaccacctgcgcaagaagctggtggacagcaccgacaaggccgacctgcgcctgatctacct
ggccctggcccacatgatcaagttccgcggccacttcctgatcgagggcgacctgaaccccgacaacagcgacgtggacaagctgttcatccagctggtgcagacctacaaccagctgttcgaggagaacccca
tcaacgccagcggcgtggacgccaaggccatcctgagcgcccgcctgagcaagagccgccgcctggagaacctgatcgcccagctgcccggcgagaagaagaacggcctgttcggcaacctgatcgccctga
gcctgggcctgacccccaacttcaagagcaacttcgacctggccgaggacgccaagctgcagctgagcaaggacacctacgacgacgacctggacaacctgctggcccagatcggcgaccagtacgccgacct
gttcctggccgccaagaacctgagcgacgccatcctgctgagcgacatcctgcgcgtgaacaccgagatcaccaaggcccccctgagcgccagcatgatcaagcgctacgacgagcaccaccaggacctgacc
ctgctgaaggccctggtgcgccagcagctgcccgagaagtacaaggagatcttcttcgaccagagcaagaacggctacgccggctacatcgacggcggcgccagccaggaggagttctacaagttcatcaagc
ccatcctggagaagatggacggcaccgaggagctgctggtgaagctgaaccgcgaggacctgctgcgcaagcagcgcaccttcgacaacggcagcatccccaccagatccacctgggcgagctgcacgccat
cctgcgccgccaggaggacttctaccccttcctgaaggacaaccgcgagaagatcgagaagatcctgaccttccgcatcccctactacgtgggccccctggcccgcggcaacagccgcttcgcctggatgacccg
caagagcgaggagaccatcaccccctggaacttcgaggaggtggtggacaagggcgccagcgcccagagcttcatcgagcgcatgaccaacttcgacaagaacctgcccaacgagaaggtgctgcccaagca
cagcctgctgtacgagtacttcaccgtgtacaacgagctgaccaaggtgaagtacgtgaccgagggcatgcgcaagcccgccttcctgagcggcgagcagaagaaggccatcgtggacctgctgttcaagacca
accgcaaggtgaccgtgaagcagctgaaggaggactacttcaagaagatcgagtgcttcgacagcgtggagatcagcggcgtggaggaccgcttcaacgccagcctgggcacctaccacgacctgctgaagat
catcaaggacaaggacttcctggacaacgaggagaacgaggacatcctggaggacatcgtgctgaccctgaccctgttcgaggaccgcgagatgatcgaggagcgcctgaagacctacgcccacctgttcgac
gacaaggtgatgaagcagctgaagcgccgccgctacaccggctggggccgcctgagccgcaagcttatcaacggcatccgcgacaagcagagcggcaagaccatcctggacttcctgaagagcgacggcttc
gccaaccgcaacttcatgcagctgatccacgacgacagcctgaccttcaaggaggacatccagaaggcccaggtgagcggccagggcgacagcctgcacgagcacatcgccaacctggccggcagcccgcc
atcaagaagggcatcctgcagaccgtgaaggtggtggacgagctggtgaaggtgatgggccgccacaagcccgagaacatcgtgatcgagatggcccgcgagaaccagaccacccagaagggccagaaga
acagccgcgagcgcatgaagcgcatcgaggagggcatcaaggagctgggcagccagatcctgaaggagcaccccgtggagaacacccagctgcagaacgagaagctgtacctgtactacctgcagaacggc
cgcgacatgtacgtggaccaggagctggacatcaaccgcctgagcgactacgacgtggacGCcatcgtgccccagagcttcctgaaggacgacagcatcgacaacaaggtgctgacccgcagcgacaagaa
ccgcggcaagagcgacaacgtgcccagcgaggaggtggtgaagaagatgaagaactactggcgccagctgctgaacgccaagctgatcacccagcgcaagttcgacaacctgaccaaggccgagcgcggcg
gcctgagcgagctggacaaggccggcttcatcaagcgccagctggtggagacccgccagatcaccaagcacgtggcccagatcctggacagccgcatgaacaccaagtacgacgagaacgacaagctgatcc
gcgaggtgaaggtgatcaccctgaagagcaagctggtgagcgacttccgcaaggacttccagttctacaaggtgcgcgagatcaacaactaccaccacgcccacgacgcctacctgaacgccgtggtgggcac
cgccctgatcaagaagtaccccaagctggagagcgagttcgtgtacggcgactacaaggtgtacgacgtgcgcaagatgatcgccaagagcgagcaggagatcggcaaggccaccgccaagtacttcttctac
agcaacatcatgaacttcttcaagaccgagatcaccctggccaacggcgagatccgcaagcgccccctgatcgagaccaacggcgagaccggcgagatcgtgtgggacaagggccgcgacttcgccaccgtgc
gcaaggtgctgagcatgccccaggtgaacatcgtgaagaagaccgaggtgcagaccggcggcttcagcaaggagagcatcctgcccaagcgcaacagcgacaagctgatcgcccgcaagaaggactgggac
cccaagaagtacggcggcttcgacagccccaccgtggcctacagcgtgctggtggtggccaaggtggagaagggcaagagcaagaagctgaagagcgtgaaggagctgctgggcatcaccatcatggagcgc
agcagcttcgagaagaaccccatcgacttcctggaggccaagggctacaaggaggtgaagaaggacctgatcatcaagctgcccaagtacagcctgttcgagctggagaacggccgcaagcgcatgctggcc
agcgccggcgagctgcagaagggcaacgagctggccctgcccagcaagtacgtgaacttcctgtacctggccagccactacgagaagctgaagggcagccccgaggacaacgagcagaagcagctgttcgtg

gagcagcacaagcactacctggacgagatcatcgagcagatcagcgagttcagcaagcgcgtgatcctggccgacgccaacctggacaaggtgctgagcgcctacaacaagcaccgcgacaagcccatccgc
gagcaggccgagaacatcatccacctgttcaccctgaccaacctgggcgcccccgccgccttcaagtacttcgacaccaccatcgaccgcaagcgctacaccagcaccaaggaggtgctggacgccaccctgat
ccaccagagcatcaccggtctgtacgagacccgcatcgacctgagccagctgggcggcgacggcggctccggacctccaaagaaaaagagaaaagtataccccctacgacgtgcccgactacgcctgttaagcg
gccgcactcgagcaccaccaccaccaccactgagatccggctgctaacaaagcccgaaaggaagctgagttggctgctgccaccgctgagcaataactagcataaccccttggggcctctaaacgggtcttgag
gggttttttgctgaaaggaggaactatatccggattggcgaatgggacgcgccctgtagcggcgcattaagcgcggcgggtgtggtggttacgcgcagcgtgaccgctacacttgccagcgccctagcgcccgct
cctttcgctttcttcccttcctttctcgccacgttcgccggctttccccgtcaagctctaaatcgggggctccctttagggttccgatttagtgctttacggcacctcgaccccaaaaaacttgattagggtgatggttca
cgtagtgggccatcgccctgatagacggtttttcgccctttgacgttggagtccacgttctttaatagtggactcttgttccaaactggaacaacactcaaccctatctcggtctattcttttgatttataagggattttg
ccgatttcggcctattggttaaaaaatgagctgatttaacaaaaa

>7_SpdCas9-VirD2-Mutated

Ccaccctgaattgactctcttccgggcgctatcatgccataccgcgaaaggttttgcgccattcgatggtgtccgggatctcgacgctctcccttatgcgactcctgcattaggaagcagcccagtagtaggttgag
gccgttgagcaccgccgccgcaaggaatggtgcatgcaaggagatggcgcccaacagtcccccggccacggggcctgccaccatacccacgccgaaacaagcgctcatgagcccgaagtggcgagcccgatc
ttccccatcggtgatgtcggcgatataggcgccagcaaccgcacctgtggcgccggtgatgccggccacgatgcgtccggcgtagaggatcgagatctcgatcccgcgaaattaatacgactcactataggggа
attgtgagcggataacaattcccctctagaaataattttgtttaactttaagaaggagatataccatgggcagcagccatcatcatcatcatcacagcagcggcctggtgccgcgcggcagccatatggctagcat
gactggtggacagcaaatgggtcgcggatccgaattcgagctccgtcgacaagcttgcggccgcatggacaagaagtacagcatcggcctggCTatcggtaccaacagcgtgggctgggccgtgatcaccgac
gagtacaaggtgcccagcaagaagttcaaggtgctgggcaacaccgaccgccacagcatcaagaagaacctgatcggcgccctgctgttcgacagcggcgagaccgccgaggccacccgcctgaagcgcacc
gcccgccgccgctacacccgccgcaagaaccgcatctgctacctgcaggagatcttcagcaacgagatggccaaggtggacgacagcttcttccaccgcctggaggagagcttcctggtggaggaggacaaga
agcacgagcgccaccccatcttcggcaacatcgtggacgaggtggcctaccacgagaagtaccccaccatctaccacctgcgcaagaagctggtggacagcaccgacaaggccgacctgcgcctgatctacct
ggccctggcccacatgatcaagttccgcggccacttcctgatcgagggcgacctgaaccccgacaacagcgacgtggacaagctgttcatccagctggtgcagacctacaaccagctgttcgaggagaacccca
tcaacgccagcggcgtggacgccaaggccatcctgagcgcccgcctgagcaagagccgccgcctggagaacctgatcgcccagctgcccggcgagaagaagaacggcctgttcggcaacctgatcgccctga
gcctgggcctgacccccaacttcaagagcaacttcgacctggccgaggacgccaagctgcagctgagcaaggacacctacgacgacgacctggacaacctgctggcccagatcggcgaccagtacgccgacct
gttcctggccgccaagaacctgagcgacgccatcctgctgagcgacatcctgcgcgtgaacaccgagatcaccaaggcccccctgagcgccagcatgatcaagcgctacgacgagcaccaccaggacctgacc
ctgctgaaggccctggtgcgccagcagctgcccgagaagtacaaggagatcttcttcgaccagagcaagaacggctacgccggctacatcgacggcggcgccagccaggaggagttctacaagttcatcaagc
ccatcctggagaagatggacggcaccgaggagctgctggtgaagctgaaccgcgaggacctgctgcgcaagcagcgcaccttcgacaacggcagcatcccccaccagatccacctgggcgagctgcacgccat
cctgcgccgccaggaggacttctaccccttcctgaaggacaaccgcgagaagatcgagaagatcctgaccttccgcatcccctactacgtgggccccctggcccgcggcaacagccgcttcgcctggatgacccg
caagagcgaggagaccatcaccccctggaacttcgaggaggtggtggacaagggcgccagcgcccagagcttcatcgagcgcatgaccaacttcgacaagaacctgcccaacgagaaggtgctgcccaagca
cagcctgctgtacgagtacttcaccgtgtacaacgagctgaccaaggtgaagtacgtgaccgagggcatgcgcaagcccgccttcctgagcggcgagcagaagaaggccatcgtggacctgctgttcaagacca
accgcaaggtgaccgtgaagcagctgaaggaggactacttcaagaagatcgagtgcttcgacagcgtggagatcagcggcgtggaggaccgcttcaacgccagcctgggcacctaccacgacctgctgaagat
catcaaggacaaggacttcctggacaacgaggagaacgaggacatcctggaggacatcgtgctgaccctgaccctgttcgaggaccgcgagatgatcgaggagcgcctgaagacctacgcccacctgttcgac
gacaaggtgatgaagcagctgaagcgccgccgctacaccggctggggccgcctgagccgcaagcttatcaacggcatccgcgacaagcagagcggcaagaccatcctggacttcctgaagagcgacggcttc
gccaaccgcaacttcatgcagctgatccacgacgacagcctgaccttcaaggaggacatccagaaggcccaggtgagcggccagggcgacagcctgcacgagcacatcgccaacctggccggcagccccgcc
atcaagaagggcatcctgcagaccgtgaaggtggtggacgagctggtgaaggtgatgggccgccacaagcccgagaacatcgtgatcgagatggcccgcgagaaccagaccacccagaagggccagaaga
acagccgcgagcgcatgaagcgcatcgaggagggcatcaaggagctgggcagccagatcctgaaggagcaccccgtggagaacacccagctgcagaacgagaagctgtacctgtactacctgcagaacggc
cgcgacatgtacgtggaccaggagctggacatcaaccgcctgagcgactacgacgtggacGCcatcgtgccccagagcttcctgaaggacgacagcatcgacaacaaggtgctgacccgcagcgacaagaa
ccgcggcaagagcgacaacgtgcccagcgaggaggtggtgaagaagatgaagaactactggcgccagctgctgaacgccaagctgatcacccagcgcaagttcgacaacctgaccaaggccgagcgcggcg
gcctgagcgagctggacaaggccggcttcatcaagcgccagctggtggagacccgccagatcaccaagcacgtggcccagatcctggacagccgcatgaacaccaagtacgacgagaacgacaagctgatcc
gcgaggtgaaggtgatcaccctgaagagcaagctggtgagcgacttccgcaaggacttccagttctacaaggtgcgcgagatcaacaactaccaccacgcccacgacgcctacctgaacgccgtggtgggcac
cgccctgatcaagaagtaccccaagctggagagcgagttcgtgtacggcgactacaaggtgtacgacgtgcgcaagatgatcgccaagagcgagcaggagatcggcaaggccaccgccaagtacttcttctac
agcaacatcatgaacttcttcaagaccgagatcaccctggccaacggcgagatccgcaagcgccccctgatcgagaccaacggcgagaccggcgagatcgtgtgggacaagggccgcgacttcgccaccgtgc
gcaaggtgctgagcatgccccaggtgaacatcgtgaagaagaccgaggtgcagaccggcggcttcagcaaggagagcatcctgcccaagcgcaacagcgacaagctgatcgcccgcaagaaggactgggac
cccaagaagtacggcggcttcgacagccccaccgtggcctacagcgtgctggtggtggccaaggtggagaagggcaagagcaagaagctgaagagcgtgaaggagctgctgggcatcaccatcatggagcgc
agcagcttcgagaagaaccccatcgacttcctggaggccaagggctacaaggaggtgaagaaggacctgatcatcaagctgcccaagtacagcctgttcgagctggagaacggccgcaagcgcatgctggcc
agcgccggcgagctgcagaagggcaacgagctggccctgcccagcaagtacgtgaacttcctgtacctggccagccactacgagaagctgaagggcagccccgaggacaacgagcagaagcagctgttcgtg
gagcagcacaagcactacctggacgagatcatcgagcagatcagcgagttcagcaagcgcgtgatcctggccgacgccaacctggacaaggtgctgagcgcctacaacaagcaccgcgacaagcccatccgc
gagcaggccgagaacatcatccacctgttcaccctgaccaacctgggcgcccccgccgccttcaagtacttcgacaccaccatcgaccgcaagcgctacaccagcaccaaggaggtgctggacgccaccctgat
ccaccagagcatcaccggtctgtacgagacccgcatcgacctgagccagctgggcggcgacggcggctccggacctccaaagaaaaagagaaaagtataccccctacgacgtgcccgactacgcctgtggagg
cggtggatctggcggtggcggaagtgggggaggtggaagtggtaccatgcccgatcgagctcaagttatcattcgcattgtgccgggaggtggcaccaagacccttcaacaaattatcaatcagttggagtatct
atcccggaagggcaggctggagctgcagcgttcagcccgacatctcgatattcccctgccaccggatcaaatccacgaacttgcccgaagctgggttcaagagactggaacttatgacgaaagtcagccagacg
aggaaaggcaacaggagttgaccacccatattattgtaagcttccccgccggtacaagccaggtagcggcttatgcggcgagccgggagtgggcagccgagatgtttgggtcaggcgcaggggggggccgata
caactatcttacggccttccacatcgatcgcgaccacccacatctgcatgtcgtcgtcaatcggcgcgaactttaggacacggctggctgaagatatctcggcgccatccccaactgaattacgacgccctgcgc
ataaagatggccgagatttcacttcgtcatggcattgccctcgatgcgagccgacgagcagaacgtggcatcaccgagcggccgatcacttatgcccaatatcggcgccttgagcgggagcaggctcgccaaat
ccgtttcgaagacgcggatttggaacagtcgtcgccgcaaggagatcatccagaattcagccaacctttcgatacatccccatttgaagcatccgcgggcggaccggaggacatgcctcggcccaacaatcggc
agaatgagtcgcaagttcatctccaggagccagctggtgtcagcaacgaagccggtgtccttgtgcgggttgcattggagacggagcgccttgctcaaccattcgtttccgaaaccattctcgcggacgacatagg
gagcggctcttcgcgtgttgccgagggccgtgtggagagcgcaaaccgcactcccgatattcctcgcgcagcaactgaagctgccacgcacacgacacacgaccggcagcggcgtgcaaagcgtcctcatgatg
acgacggagggccgagtggagcaaaacgtgtgacattggaaggcatcgcggttgggccccaggcgaacgccggcgaacaggatggcagtagtggccccttagtacggcaagctggaacgtctcggccatctc
caccgacggccacgacgcgggccagcaccgcaaccgattcattgtctgctacagcccacctccagcaacggagaggtgtcctttcaaagcgtccgcgtgaagatgatgatggagaaccgagtgaacgcaaacg
cgagagagatgagcgcagcaaggacgggcgtgggggaaataggagataataagcggccgcactcgagcaccaccaccaccaccactgagatccggctgctaacaaagcccgaaaggaagctgagttggctg
ctgccaccgctgagcaataactagcataaccccttggggcctctaaacgggtcttgaggggttttttgctgaaaggaggaactatatccggattggcgaatgggacgcgccctgtagcggcgcattaagcgcggc
gggtgtggtggttacgcgcagcgtgaccgctacacttgccagcgccctagcgcccgctcctttcgctttcttcccttcctttctcgccacgttcgccggctttccccgtcaagctctaaatcgggggctccctttagggt
tccgatttagtgctttacggcacctcgaccccaaaaaacttgattagggtgatggttcacgtagtgggccatcgccctgatagacggtttttcgccctttgacgttggagtccacgttctttaatagtggactcttgttc
caaactggaacaacactcaaccctatctcggtctattcttttgatttataagggattttgccgatttcggcctattggttaaaaaatgagctgatttaacaaaaatttaacgcgaattttaacaaaatattaacgctta
caatttaggtggcacttttcggggaaatgtgcgcggaacccctatttgtttatttttctaaatacattcaaatatgtatccgctcatgaattaattcttagaaaaactcatcgagcatcaaatgaaactgcaatttatt
catatcaggattatcaataccatatttttgaaaaagccgtttctgtaatgaaggagaaaactcaccgaggcagttccataggatggcaagatcctggtatcggtctgcgattccgactcgtccaacatcaatacaa
cctattaatttcccctcgtcaaaaataaggttatcaagtgagaaatcaccatgagtgacgactgaatccggtgagaatggcaaaagtttatgcatttctttccagacttgttcaacaggccagccattacgctcgtc
atcaaaatcactcgcatcaaccaaaccgttattcattcgtgattgcgcctgagcgagacgaaatacgcgatcgctgttaaaaggacaattacaaacaggaatcgaatgcaaccggcgcaggaacactgccagc

gcatcaacaatattttcacctgaatcaggatattcttctaatacctggaatgctgttttcccggggatcgcagtggtgagtaaccatgcatcatcaggagtacggataaaatgcttgatggtcggaagaggcataa
attccgtcagccagtttagtctgaccatctcatctgtaacatcattggcaacgctacctttgccatgtttcagaaacaactctggcgcatcgggcttcccatacaatcgatagattgtcgcacctgattgcccgacatt
atcgcgagcccatttatacccatataaatcagcatccatgttggaatttaatcgcggcctagagcaagacgtttcccgttgaatatggctcataacaccccttgtattactgtttatgtaagcagacagttttattgtt
catgaccaaaatcccttaacgtgagttttcgttccactgagcgtcagaccccgtagaaaagatcaaaggatcttcttgagatcctttttttctgcgcgtaatctgctgcttgcaaacaaaaaaaccaccgctaccag
cggtggtttgtttgccggatcaagagccaccaactctttttccgaaggtaactggcttcagcagagcgcagataccaaatactgtccttctagtgtagccgtagttaggccaccacttcaagaactctgtagcaccg
cctacatacctcgctctgctaatcctgttaccagtggctgctgccagtggcgataagtcgtgtcttaccgggttggactcaagacgatagttaccggataaggcgcagcggtcgggctgaacggggggttcgtgca
cacagcccagcttggagcgaacgacctacaccgaactgagatacctacagcgtgagctatgagaaagcgccacgcttcccgaagggagaaaggcggacaggtatccggtaagcggcagggtcggaacagga
gagcgcacgagggagcttccagggggaaacgcctggtatctttatagtcctgtcgggtttcgccacctctgacttgagcgtcgatttttgtgatgctcgtcaggggggcggagcctatggaaaaacgccagcaacg
cggcctttttacggttcctggccttttgctggccttttgctcacatgttctttcctgcgttatcccctgattctgtggataaccgtattaccgcctttgagtgagctgataccgctcgccgcagccgaacgaccgagcgca
gcgagtcagtgagcgaggaagcggaagagcgcctgatgcggtattttctccttacgcatctgtgcggtatttcacaccgcaatggtgcactctcagtacaatctgctctgatgccgcatagttaagccagtatacac
tccgctatcgctacgtgactgggtcatggctgcgccccgacacccgccaacacccgctgacgcgccctgacgggcttgtctgctcccggcatccgcttacagacaagctgtgaccgtctccgggagctgcatgtgtc
agaggttttcaccgtcatcaccgaaacgcgcgaggcagctgcggtaaagctcatcagcgtggtcgtgaagcgattcacagatgtctgcctgttcatccgcgtccagctcgttgagtttctccagaagcgttaatgtc
tggcttctgataaagcgggccatgttaagggcggtttttttcctgtttggtcactgatgcctccgtgtaagggggatttctgttcatgggggtaatgataccgatgaaacgagagaggatgctcacgatacgggttact
gatgatgaacatgcccggttactggaacgttgtgagggtaaacaactggcggtatggatgcggcgggaccagagaaaaatcactcagggtcaatgccagcgcttcgttaatacagatgtaggtgttccacaggg
tagccagcagcatcctgcgatgcagatccggaacataatggtgcagggcgctgacttccgcgtttccagactttacgaaacacggaaaccgaagaccattcatgttgttgctcaggtcgcagacgttttgcagca
gcagtcgcttcacgttcgctcgcgtatcggtgattcattctgctaaccagtaaggcaaccccgccagcctagccgggtcctcaacgacaggagcacgatcatgcgcacccgtggggccgccatgccggcgataat
ggcctgcttctcgccgaaacgtttggtggcgggaccagtgacgaaggcttgagcgagggcgtgcaagattccgaataccgcaagcgacaggccgatcatcgtcgcgctccagcgaaagcggtcctcgccgaaa
atgacccagagcgctgccggcacctgtcctacgagttgcatgataaagaagacagtcataagtgcggcgacgatagtcatgccccgcgcccaccggaaggagctgactgggttgaaggctctcaagggcatcg
gtcgagatcccggtgcctaatgagtgagctaacttacattaattgcgttgcgctcactgcccgctttccagtcgggaaacctgtcgtgccagctgcattaatgaatcggccaacgcgcggggagaggcggtttgcg
tattgggcgccagggtggtttttcttttcaccagtgagacgggcaacagctgattgcccttcaccgcctggccctgagagagttgcagcaagcggtccacgctggtttgccccagcaggcgaaaatcctgtttgatg
gtggttaacggcgggatataacatgagctgtcttcggtatcgtcgtatcccactaccgagatatccgcaccaacgcgcagcccggactcggtaatggcgcgcattgcgcccagcgccatctgatcgttggcaacca
gcatcgcagtgggaacgatgccctcattcagcatttgcatggtttgttgaaaaccggacatggcactccagtcgccttcccgttccgctatcggctgaatttgattgcgagtgagatatttatgccagccagccaga
cgcagacgcgccgagacagaacttaatgggcccgctaacagcgcgatttgctggtgacccaatgcgaccagatgctccacgcccagtcgcgtaccgtcttcatgggagaaaataatactgttgatgggtgtctgg
tcagagacatcaagaaataacgccggaacattagtgcaggcagcttccacagcaatggcatcctggtcatccagcggatagttaatgatcagcccactgacgcgttgcgcgagaagattgtgcaccgccgcttta
caggcttcgacgccgcttcgttctaccatcgacaccaccacgctggcacccagttgatcggcgcgagatttaatcgccgcgacaatttgcgacggcgcgtgcagggccagactggaggtggcaacgccaatcag
caacgactgtttgcccgccagttgttgtgccacgcggttgggaatgtaattcagctccgccatcgccgcttccactttttcccgcgttttcgcagaaacgtggctggcctggttcaccacgcgggaaacggtctgata
agagacaccggcatactctgcgacatcgtataacgttactggtttcacattca
